# TGF-β-induced cell motility requires downregulation of *ARHGAP*s to sustain Rac1 activity

**DOI:** 10.1016/j.jbc.2021.100545

**Published:** 2021-03-17

**Authors:** Mitsuyoshi Motizuki, Daizo Koinuma, Takashi Yokoyama, Yuka Itoh, Chiho Omata, Kohei Miyazono, Masao Saitoh, Keiji Miyazawa

**Affiliations:** 1 Department of Biochemistry, Graduate School of Medicine, University of Yamanashi, Yamanashi, Japan; 2 Department of Molecular Pathology, Graduate School of Medicine, The University of Tokyo, Tokyo, Japan; 3 Center for Medical Education and Science, Graduate School of Medicine, University of Yamanashi, Yamanashi, Japan

**Keywords:** Smad, cell motility, Rac1, ARHGAP, EMT, epithelial–mesenchymal transition, GAP, GTPase-activating protein, GEF, guanine nucleotide exchange factor, PI3K, phosphoinositide 3-kinase, TGF-β, transforming growth factor-β

## Abstract

Transforming growth factor-β (TGF-β) signaling promotes cancer progression. In particular, the epithelial–mesenchymal transition (EMT) induced by TGF-β is considered crucial to the malignant phenotype of cancer cells. Here, we report that the EMT-associated cellular responses induced by TGF-β are mediated by distinct signaling pathways that diverge at Smad3. By expressing chimeric Smad1/Smad3 proteins in *SMAD3* knockout A549 cells, we found that the β4 region in the Smad3 MH1 domain is essential for TGF-β-induced cell motility, but is not essential for other EMT-associated responses including epithelial marker downregulation. TGF-β was previously reported to enhance cell motility by activating Rac1 *via* phosphoinositide 3-kinase. Intriguingly, TGF-β-dependent signaling mediated by Smad3's β4 region causes the downregulation of multiple mRNAs that encode GTPase activating proteins that target Rac1 (*ARHGAP*s), thereby attenuating Rac1 inactivation. Therefore, two independent pathways downstream of TGF-β type I receptor contribute cooperatively to sustained Rac1 activation, thereby leading to enhanced cell motility.

Transforming growth factor-β (TGF-β) is a pleiotropic cytokine that regulates a wide variety of cellular processes ranging from embryogenesis to adult tissue homeostasis. TGF-β signals principally through Smad proteins. Smad2 and Smad3, termed receptor-regulated Smads, are phosphorylated at their C termini by TGF-β type I receptor activated by ligand stimulation. Thereafter, phosphorylated Smad2 and Smad3 form a heterotrimeric complex with Smad4 before they are translocated into the nucleus. The Smad complex subsequently regulates gene expression by binding to genomic regulatory regions in cooperation with Smad-binding transcription factors and coregulatory proteins (1).

Smad-binding transcription factors are known collectively as “Smad cofactors.” Smad cofactors are thought to assist in selective as well as stable binding of Smad proteins to genomic DNA, thereby enabling context-dependent Smad-mediated transcription in various target cells (2). Alternatively, activated Smad proteins can regulate gene expression indirectly by interacting physically with other transcription factors, thereby either repressing or derepressing their functions (3, 4, 5). Smad proteins also promote miRNA processing upon their activation by TGF-β (6). In addition to these Smad-dependent pathways, TGF-β can transmit signals independently of Smad proteins. Examples of such pathways include Shc-mediated activation of the Ras-MAP kinase pathway (7) and TRAF4/6-mediated activation of p38 MAP kinases, c-Jun N-terminal kinases, and phosphoinositide 3-kinase (PI3K) (8, 9).

TGF-β has two opposing effects on cancer progression (10). In normal cells, TGF-β inhibits cell cycle progression and contributes to genome stability. In addition, TGF-β induces apoptosis in some cell types, thereby suppressing carcinogenesis. However, in transformed cells, TGF-β induces epithelial–mesenchymal transition (EMT) and promotes cancer cell malignancy (11). EMT is a process by which cells alter their phenotypic features from epithelial to mesenchymal and is characterized by acquisition of spindle cell morphology, destabilization of adherens junctions, actin stress fiber formation, and enhanced cell motility as well as invasiveness (12). These cellular responses are mediated primarily through the coordinated actions of EMT-associated transcription factors (EMT-TFs), each of which confers distinct phenotypes to target cells (13). EMT phenotypes can therefore be variable and sometimes “partial” depending on the EMT-TFs utilized in each context that reflects target cell types or the nature of extracellular signaling cues (EMT-TF code) (14).

To initiate EMT, TGF-β transmits signals that upregulate expression of transcription factors that drive EMT, such as Snail and ZEB1 (4, 15, 16, 17, 18, 19). However, TGF-β also activates multiple signaling pathways, including PI3K and the Rho family GTPases, which are also involved in EMT-associated cellular responses (9, 20, 21). Therefore, TGF-β-induced EMT appears to be modulated by these additional signaling pathways.

Intriguingly, there have been indications that signaling pathways that promote cell motility may diverge at the Smad proteins from those leading to upregulation of EMT-TFs. An Id-like helix-loop-helix protein, Maid, suppresses TGF-β-induced cell motility, but not other EMT-associated responses, including E-cadherin downregulation and actin stress fiber formation, in normal murine mammary gland epithelial cells (NMuMG cells) (22).

In this study, we examined signaling pathways leading to EMT-associated cellular responses using A549 human lung adenocarcinoma cells, which are known to undergo complete EMT in response to TGF-β. By expressing either wild-type or mutant Smad3 exogenously in *SMAD3*-knockout A549 cells, we observed divergence of the pathways that lead to epithelial marker downregulation and cell motility, at Smad3. We further dissected the pathways needed for TGF-β-induced cell motility. The PI3K pathway is known to play a crucial role in enhanced cell motility induced by TGF-β (23). We found that the transcription pathway mediated by the β4 region of the Smad3 MH1 domain is also essential. The PI3K pathway activates Rac1 by activating Rac-GEFs (24) while the Smad3 β4 region-mediated pathway dictates downregulation of GTPase-activating proteins that target Rac1, thereby preventing Rac1 inactivation. Therefore, two pathways downstream of TGF-β cooperatively induce sustained Rac1 activation to enhance cell motility. The findings of this study help explain why TGF-β-induced cell motility requires a Smad-dependent transcriptional program in addition to PI3K activation.

## Results

### Identification of Smad3-dependent EMT-associated cellular responses

**Figure 1:**
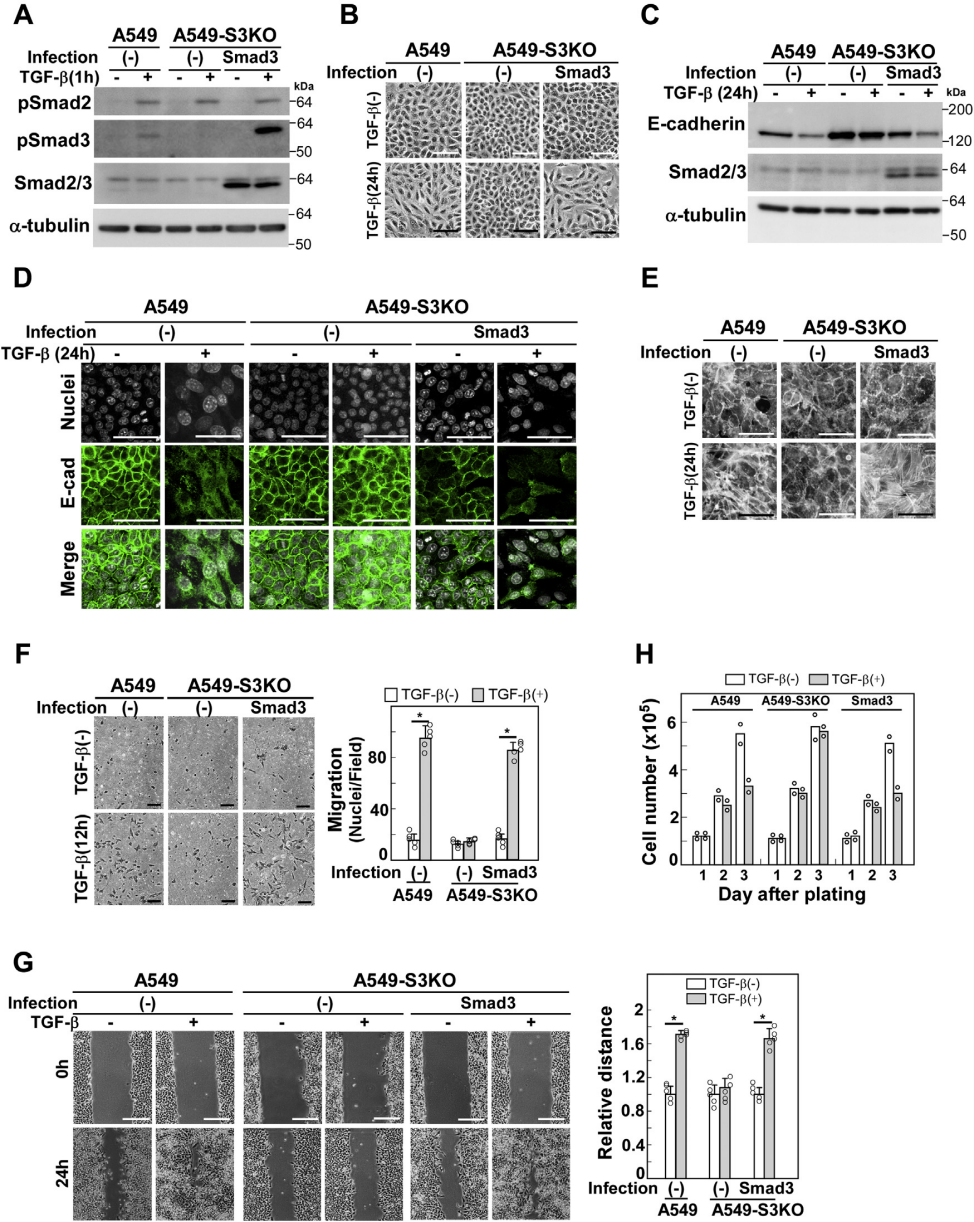
**EMT-associated cellular responses that are dependent on Smad3**. *A*, *SMAD3* knockout A549 cells (A549-S3KO) were prepared using CRISPR/Cas9-mediated genome editing. A549-S3KO cells were infected with lentivirus carrying *SMAD3* cDNA. Expression and TGF-β-induced phosphorylation of Smad3 were verified by immunoblotting with the indicated antibodies; α-tubulin was used as a loading control. *B–E*, A549 cells, A549-S3KO cells, or those expressing wild-type Smad3 were incubated in either the presence or absence of 1 ng/ml TGF-β1 for 24 h. *B*, light microscopic photographs. *C*, expression of E-cadherin was determined by immunoblotting with anti-E-cadherin; α-tubulin was used as a loading control. *D*, immunofluorescence detection of E-cadherin. *E*, formation of actin stress fibers in A549-S3KO cells expressing wild-type Smad3. F-actin was stained using Rhodamine-phalloidin. *F*, chamber migration assay or (*G*) wound healing assay of A549 cells, A549-S3KO cells, or those expressing wild-type Smad3 in either the presence or absence of 1 ng/ml TGF-β1. Quantification is shown in the right. *H*, to evaluate growth rates of A549 cells, A549-S3KO cells, or those expressing wild-type Smad3 in either the presence or absence of 1 ng/ml TGF-β1, cell numbers were counted. TGF-β was added at day1. Scale bars: 10 μm (*B* and *D*–*F*) and 200 μm (*G*). Error bars represent SD (n = 5 for *F*, *G*). *p* values were determined by Student's *t*-test. ∗*p* < 0.01. One representative result from two independent experiments is shown (*F–H*).

We previously established *SMAD3* knockout A549 (A549-S3KO) cells using CRISPR/Cas9-mediated genome editing (5). The endogenous expression and TGF-β-induced phosphorylation of Smad3 were abrogated while those of Smad2 were not, thus ensuring specific effects of the knockout (Fig. 1*A*). We subsequently examined EMT-associated cellular responses in A549-S3KO cells following TGF-β stimulation. TGF-β failed to induce cell morphological changes, downregulation of the epithelial marker E-cadherin, actin stress fiber formation, and enhanced cell motility (Fig. 1, *B*–*G*). Basal cell proliferation was not affected but TGF-β failed to induce growth inhibition in *SMAD3* knockout cells (Fig. 1*H*), suggesting that suppression of cell motility was not caused by differences in cell proliferation rate. The defective phenotype noted above was restored by lentiviral expression of Smad3 (Fig. 1, *A*–*H*), indicating that these phenotypic alterations are attributed to the loss of Smad3.

### Role of the Smad3 MH1 domain in TGF-β-induced signaling

Smad3 mediates transmission of divergent signals in response to TGF-β stimulation, utilizing its molecular surface for physical interaction with a variety of proteins and RNAs. To dissect Smad3-mediated signaling pathways that facilitate EMT-associated cellular responses, we went on to identify the regions in Smad3 involved in these responses using Smad1/3-chimeric proteins. The three-dimensional structures of the MH1 and MH2 domains of Smad1 and Smad3 have been solved and the main chains of both Smad proteins were shown to be quite similar (25, 26, 27, 28, 29). Therefore, Smad1/3 chimeric proteins have been successfully used to identify regions responsible for interaction with binding partners (30, 31, 32).

We first tried to identify region(s) in the MH2 domain responsible for EMT-associated cellular responses. We constructed a Smad1/3 chimera 3-3-1(HD/RT) that contains the MH1 domain and the linker from Smad3 and the MH2 domain from Smad1 (residues Glu-239 to Ser-425 of Smad3 were replaced by Glu-278 to Ser-465 of Smad1 with a double mutation His425Arg/Asp428Thr to allow TGF-β–dependent C-terminal phosphorylation) (33) (Fig. S1*A*). When introduced into A549-S3KO cells, 3-3-1(HD/RT) rescued cell morphological changes, downregulation of the epithelial marker E-cadherin, actin stress fiber formation, and enhanced cell motility (Fig. S1, *B–F*). Therefore, we focused instead on the MH1 domain. A Smad1/3 chimera 1-3-3, which contains the MH1 domain from Smad1 and the linker and MH2 domain from Smad3 (residues Met-1–Thr-144 of Smad3 were replaced by those of Smad1), failed to rescue any of the above cellular responses in A549-S3KO cells.

**Figure 2:**
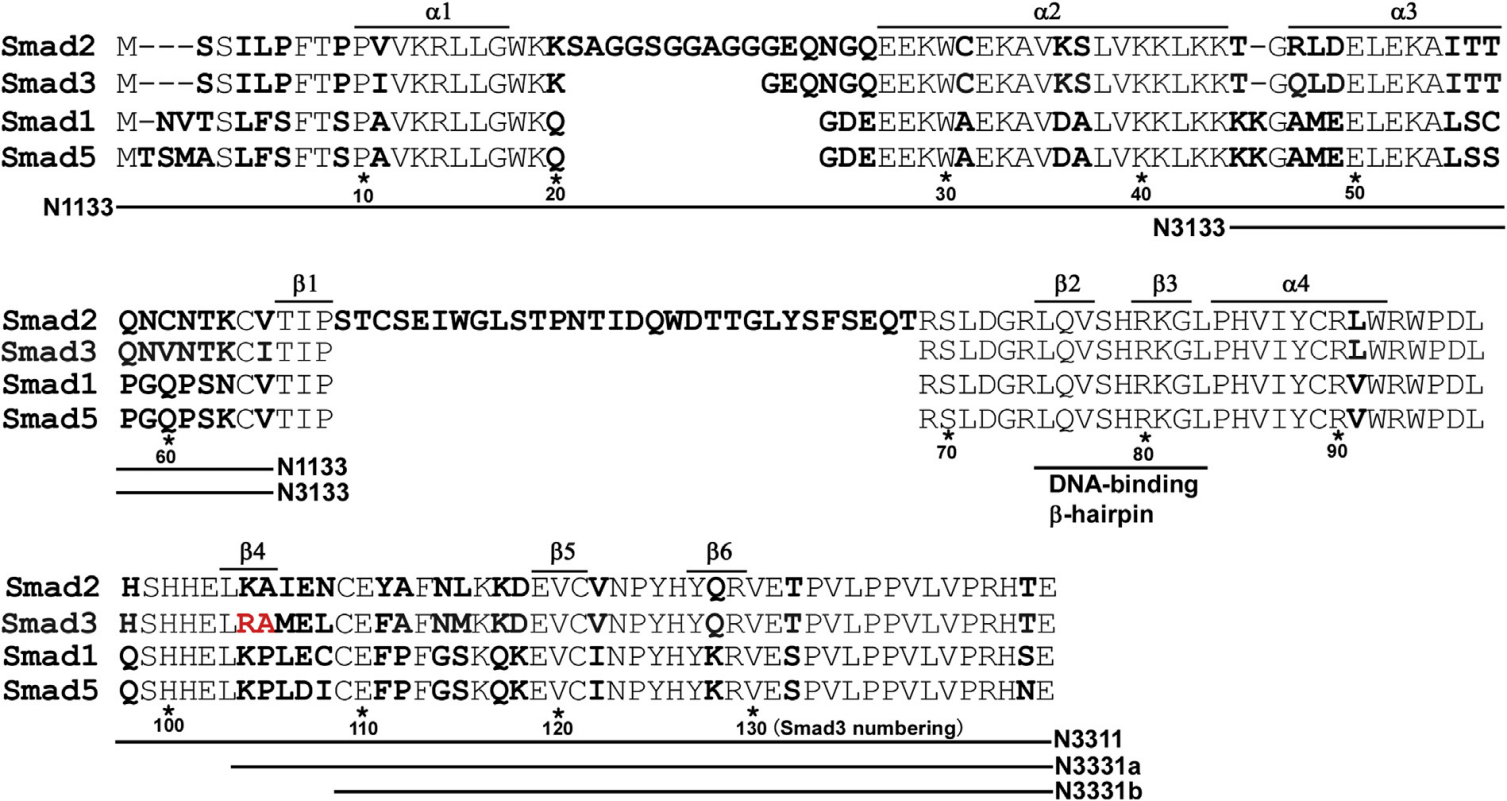
**Alignment of Smad1, Smad2, Smad3, and Smad5 protein sequences**. Diverged residues are shown in bold. Arg-104 and Ala-105 in Smad3 are shown in *red*. Smad1-derived regions in Smad1/3 chimeras are shown in *line*.

**Figure 3:**
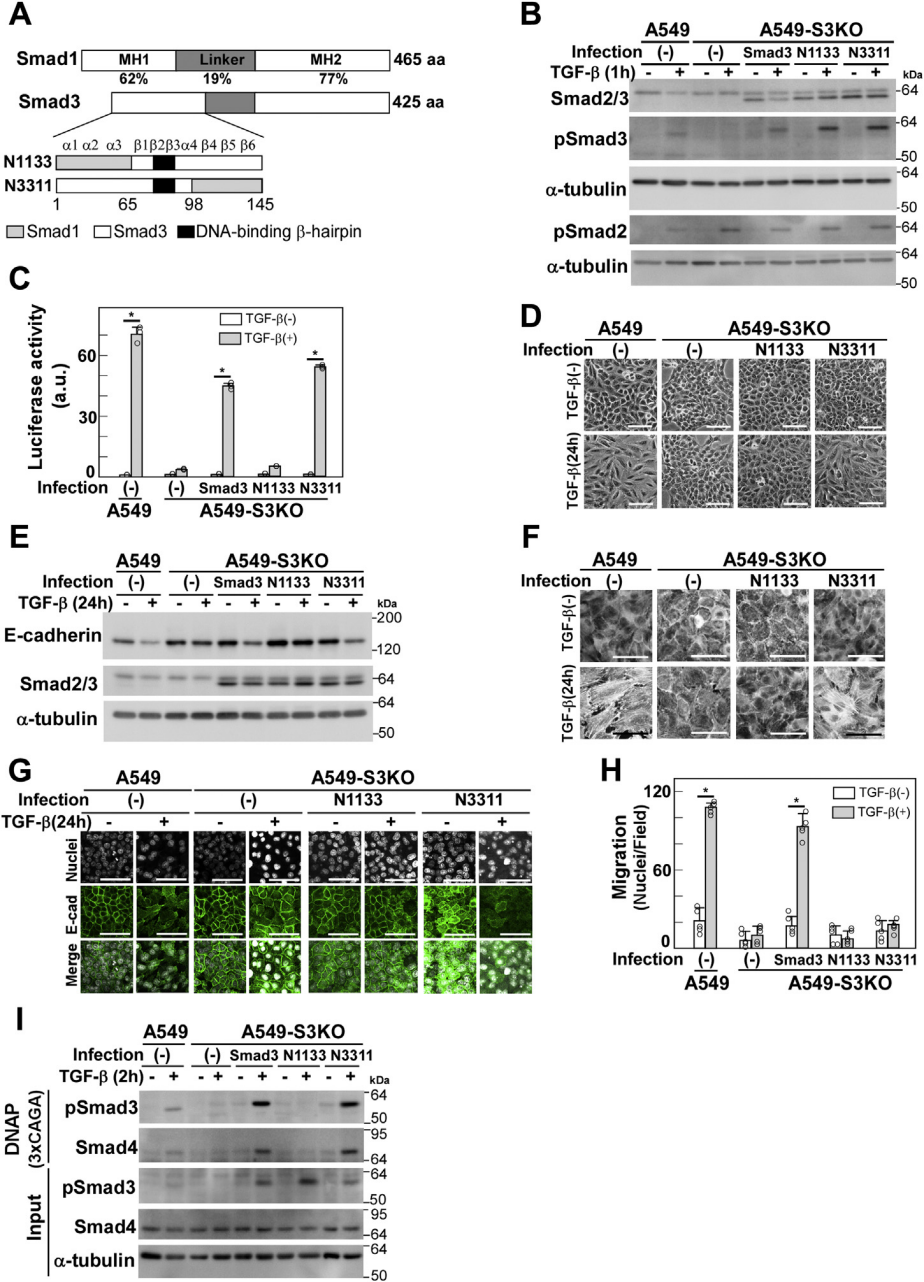
**Signaling of TGF-β-induced cell motility is transmitted through a distinct region of Smad3 from other EMT-associated responses**. *A*, schematic presentation of N1133 and N3311 chimeric proteins. N1133 (Smad3 with Met-1 to Val-65 from Smad1); N3311 (Smad3 with Gln-98 to Ser-144 from Smad1). *B*, A549-S3KO cells were infected with lentivirus carrying cDNA expressing either the N1133 or N3311 chimera. Expression and TGF-β-induced phosphorylation of N1133 and N3311 were verified by immunoblotting with indicated antibodies; α-tubulin was used as a loading control. *C*, CAGA-Luc assay of A549-S3KO cells expressing N1133 or N3311. Luciferase assay was carried out using (CAGA)_12_-MLP-Luc as a reporter plasmid. Error bars represent SD (n = 3). *D*, light microscopic images, (*E*) expression of E-cadherin detected by immunoblotting, (*F*) formation of actin stress fibers visualized by Rhodamine-phalloidin staining, (*G*) immunofluorescence labeling of E-cadherin, and (*H*) chamber migration assay in A549-S3KO cells expressing wild-type Smad3, N1133, or N3311 in either the presence or absence of 1 ng/ml TGF-β1. Scale bars: 10 μm. Error bars represent SD (n = 5). *p* values were determined by Student's *t*-test. ∗*p* < 0.01. *I*, DNA affinity precipitation assay (DNAP) was performed using biotinylated 3xCAGA as a probe. Total cell lysates (input) and proteins in cell lysates bound to the probe were analyzed by immunoblotting with indicated antibodies; α-tubulin was used as a loading control. One representative result from two independent experiments is shown (*C*, *H*, *I*).

The MH1 domains of R-Smads share a well-conserved region in their central part (Arg-69–Leu-97 for Smad3, Fig. 2) with only one divergent residue at Leu-91. The conserved region contains a DNA-binding β-hairpin that spans Leu-75–Gly-82 (26) while the divergent regions may be involved in specific binding to Smad cofactors (34, 35, 36). To identify potential residues responsible for EMT-associated cellular responses induced by TGF-β, we constructed two Smad3 mutant proteins in which either the N-terminal (from Met-1 to Ile-65) or C-terminal (from His-98 to Thr-144) region of the MH1 domain was replaced by the corresponding region from Smad1, respectively (N1133 and N3311, Figs. 2 and 3*A*). Using a lentiviral expression system, we introduced these chimeric constructs into A549-S3KO cells (Fig. 3*B*), verified that they undergo C-terminal phosphorylation following TGF-β stimulation, and observed the corresponding cellular responses.

N3311 transactivated the CAGA reporter (Fig. 3*C*) and transmitted signals leading to changes in cell morphology, E-cadherin downregulation, actin stress fiber formation, but not those that promote enhanced cell motility (Fig. 3, *D*–*H*). These findings indicate that the C-terminal region of Smad3 MH1 (His-98–Thr-144) is essential for TGF-β-enhanced cell motility (Fig. 3*H*), but not for other responses (Fig. 3, *D*–*G*). By contrast, N1133 failed to mediate the TGF-β-induced activity of (CAGA)_12_-MLP-Luc (Fig. 3*C*) and other cellular responses (Fig. 3, *D*–*H*). The N-terminal region of the MH1 domain was previously reported to be crucial for direct DNA binding of Smad3 (30). Consistently, N1133 binds poorly to the CAGA box sequence in a DNA affinity precipitation assay (Fig. 3*I*). Thus, we concluded that the N-terminal region of Smad3 MH1 domain (Met-1–Ile-65) is required for Smad3-mediated transcription activation.

### The β4 region of Smad3 is essential for TGF-β-enhanced cell motility, but not other EMT-associated cell responses

**Figure 4:**
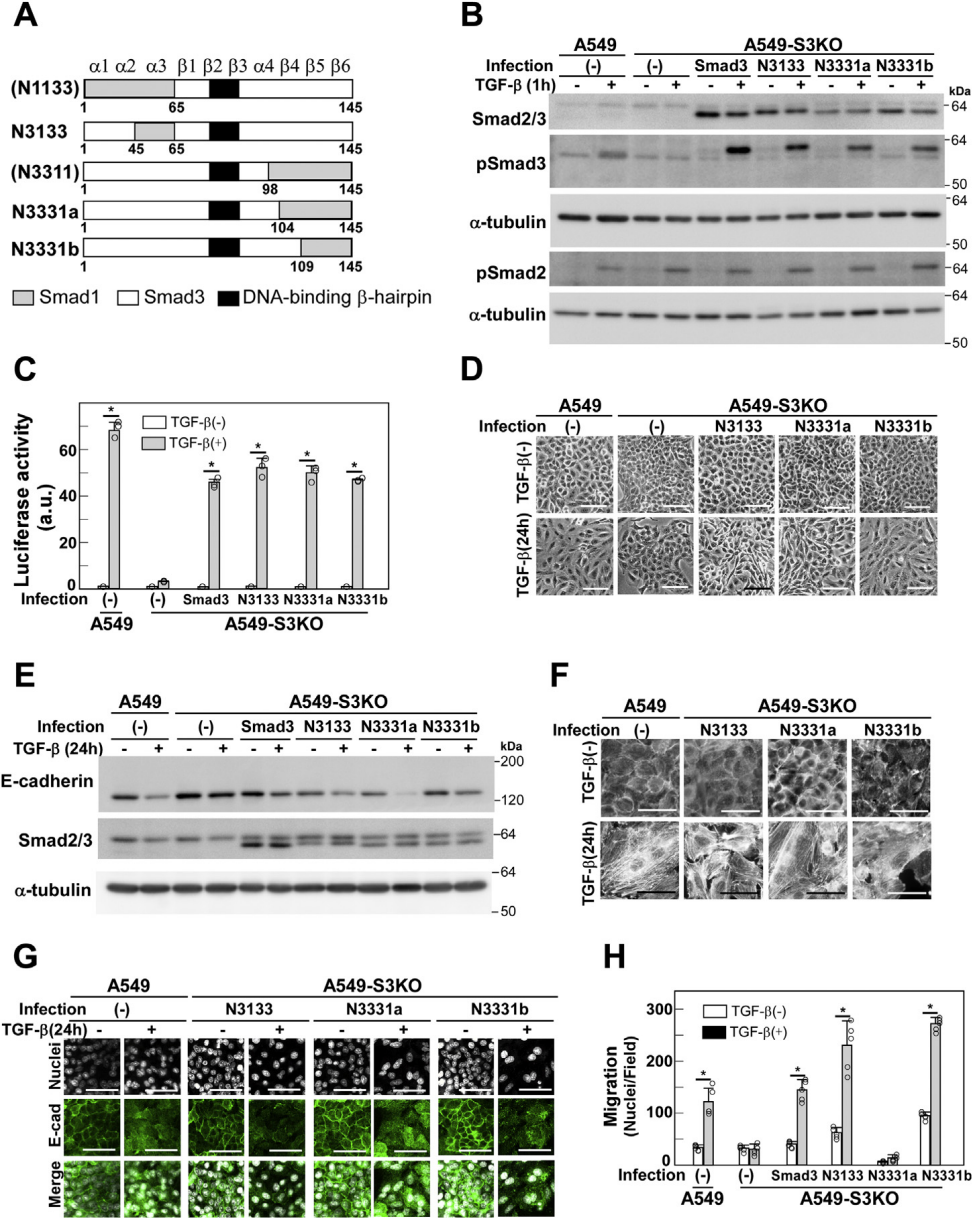
**The β4 region of Smad3 is indispensable for TGF-β-enhanced cell motility.***A*, schematic presentation of Smad1/3 chimeric proteins. N3133 (Smad3 with Lys-45 to Val-65 replacement from Smad1); N3331a (Smad3 with Lys-104 to Ser-144 replacement from Smad1); N3331b (Smad3 with Cys-109 to Ser-144 replacement from Smad1). *B*, A549-S3KO cells were infected with lentivirus carrying cDNA encoding the N3133, N3331a, or N3331b chimera. Expression and TGF-β-induced phosphorylation of N3133, N3331a, and N3331b were verified by immunoblotting with indicated antibodies; α-tubulin was used as a loading control. *C*, CAGA-Luc assay, (*D*) light microscopic images, (*E*) expression of E-cadherin detected by immunoblotting, (*F*) formation of actin stress fibers visualized by Rhodamine-phalloidin staining, (*G*) immunofluorescence labeling of E-cadherin, and (*H*) chamber migration assay in A549-S3KO cells expressing wild-type Smad3, N3133, N3331a, or N3331b in either the presence or absence of 1 ng/ml TGF-β1. Scale bars: 10 μm. Error bars represent SD (n = 3 for *C* and n = 5 for *H*). *p* values were determined by Student's *t*-test. ∗*p* < 0.01. One representative result from two independent experiments is shown (*C*, *H*).

To identify the region responsible for TGF-β-induced signaling, we constructed three additional Smad3 mutants, in which indicated amino acid residues were replaced by the corresponding residues from Smad1 (Figs. 2 and 4*A*); N3133, Thr-45–Ile-65; N3331a, Arg-104–Thr-144; and N3331b, Cys-109–Thr-144. The latter two chimeras were constructed in order to examine possible contribution of the divergent region that spans between Arg-104 and Leu-108. These chimeric constructs were introduced into A549-S3KO cells and their equivalent expression levels as well as TGF-β-induced C-terminal phosphorylation were verified experimentally (Fig. 4*B*).

**Figure 5:**
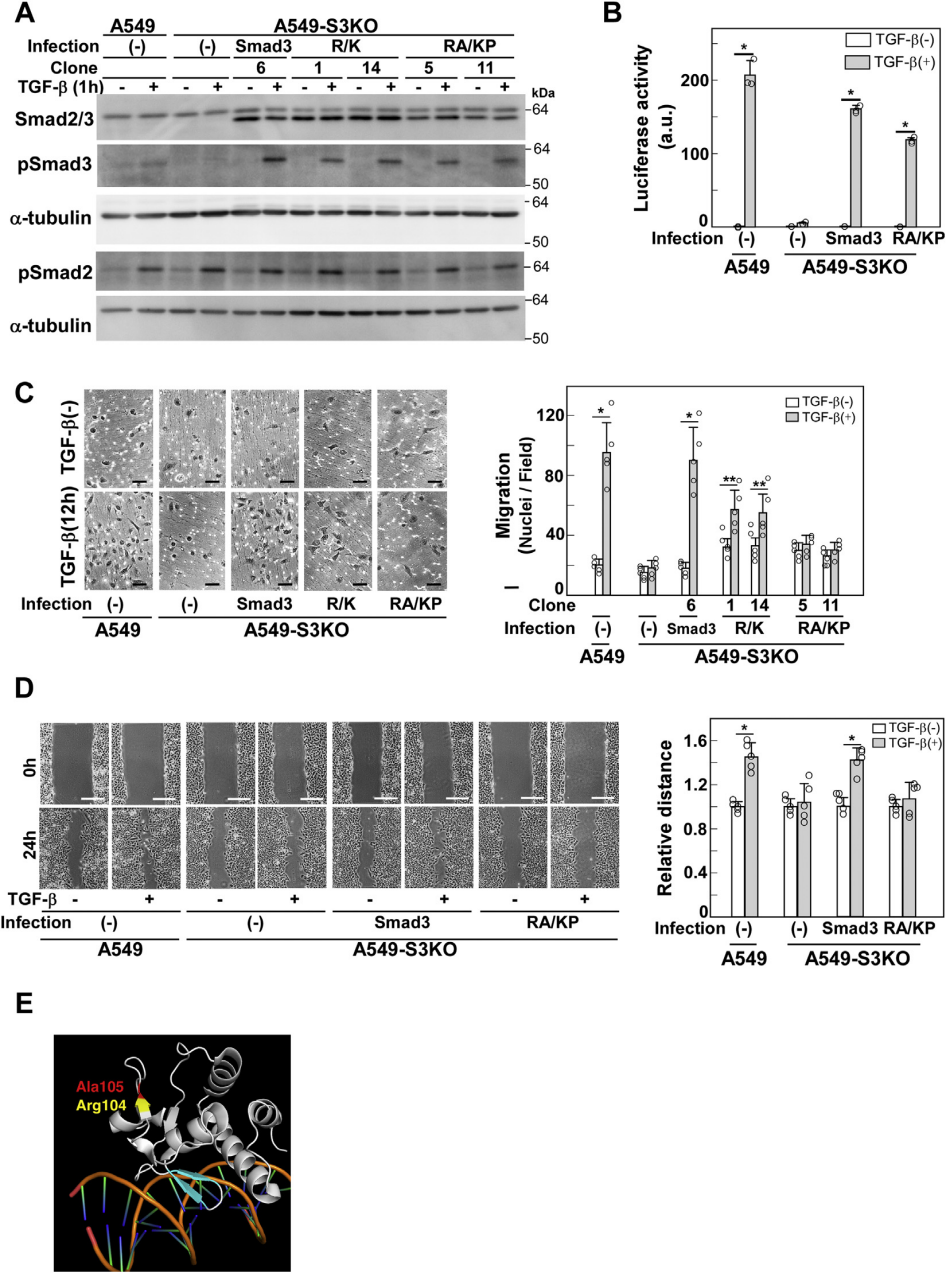
**Smad3(RA/KP) fails to transmit signals for cell motility**. A549-S3KO cells were infected with lentivirus carrying cDNA encoding either Smad3 with an Arg104Lys substitution (R/K) or the Smad3 Arg104Lys/Ala105Pro double mutant (RA/KP). *A*, expression and TGF-β-induced phosphorylation of Smad3(R/K) and Smad3(RA/KP) were verified by immunoblotting with indicated antibodies; α-tubulin was used as a loading control. *B*, CAGA-Luc assay, (*C*) chamber migration assay, and (*D*) wound healing assay in A549-S3KO cells expressing wild-type Smad3, Smad3(R/K), or Smad3(RA/KP) in either the presence or absence of 1 ng/ml TGF-β1. Scale bars: 10 μm (*C*), 200 μm (*D*). Error bars represent SD (n = 3 for *B* and n = 5 for *C*, *D*). *p* values were determined by Student's *t*-test. ∗*p* < 0.01. ∗∗*p* < 0.05. *E*, molecular model of the β4 region in the 3D structure of Smad3's MH1 domain bound to DNA (PDB ID 1OZJ). Arg-104 and Ala-105 are shown in *yellow* and *red*, respectively. The DNA-binding β-hairpin containing strands β2 and β3 is shown in cyan. Image of 3D structure was generated with PyMOL (http://pymol.org). One representative result from two independent experiments is shown (*B–D*).

N3133 was able to rescue defective activation of (CAGA)_12_-MLP-Luc reporter activity, changes in cell morphology, E-cadherin downregulation, stress fiber formation, and enhanced cell motility (Fig. 4, *C*–*H*). Thus, the residues Thr-45–Ile-65 in Smad3 can be replaced by the corresponding region in Smad1 to transmit signals leading to EMT-associated cellular responses. In particular, N3331b also mediated all of the above responses, whereas N3331a failed to rescue enhanced cell motility. Therefore, residues Arg-104–Leu-108 in Smad3 are indispensable for TGF-β-enhanced cell motility. Through mutagenic analysis, we found that Arg-104 and Ala-105 are key to discriminating Smad3 from Smad1 when transmitting signals (Fig. 2, Fig. 5, *A*–*D* and Fig. S2): Smad3 (R104 K/A105P) rescued EMT-associated cell responses other than cell motility. These two residues are located in the β4 region that is exposed to the exterior and possibly constitute a protein–protein interaction surface (Fig. 5*E*). Thereafter, we named the R104 K/A105P Smad3 double mutant Smad3(RA/KP) and used it to elucidate the signaling pathway leading to cell motility. Abilities of the Smad1/3 chimeras to support different cellular responses are summarized in Table S1.

### Smad3(RA/KP) fails to induce apparent Rac1 activation

We went on to uncover the signaling pathway mediated through the β4 region of the Smad3 MH1 domain. Cell movement requires the coordinated action of the cytoskeleton and cell adhesion molecules (37). Lamellipodia are membrane protrusions composed of actin filaments, which are formed along the leading edge of migrating cells. Confluent cultures of A549 cells were scratched and incubated with TGF-β for 16 h. Lamellipodia formation was observed in A549 cells, but only rarely in A549-S3KO cells (Fig. S3), indicating that lamellipodia formation is a Smad3-dependent process. Notably, Smad3(RA/KP) fails to rescue lamellipodia formation induced by TGF-β in A549-S3KO cells. Instead, stress fiber formation was prominent. Its failure to rescue lamellipodia formation is the likely reason why this mutant is not able to rescue cell motility induced by TGF-β.

**Figure 6:**
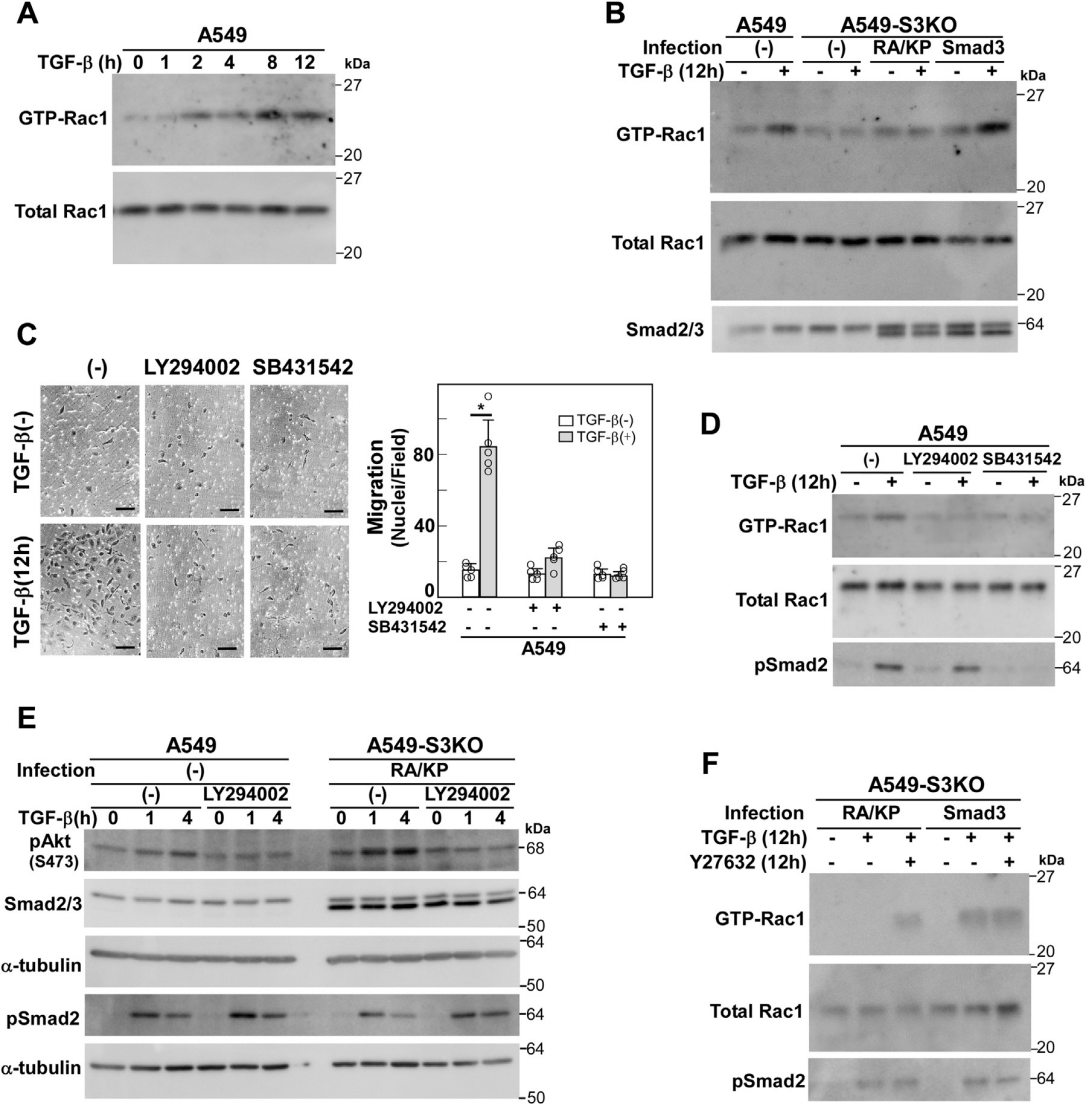
**Activation of Rac1 during TGF-β–induced motility**. *A*, time course showing Rac1 activation after TGF-β stimulation. A549 cells were stimulated with 1 ng/ml TGF-β1 for 1–12 h. The amount of active, GTP-loaded Rac1 was determined by using a GST pull-down assay. Rac1 was detected by immunoblotting. *B*, Rac1 activation assay using A549, A549-S3KO cells, or those expressing Smad3 or Smad3(RA/KP). Cells were stimulated with 1 ng/ml TGF-β1 for 12 h before harvesting. Cell lysates were subsequently prepared and subjected to a Rac1 activation assay as in *A*. *C*, chamber migration assay using A549 cells pretreated with either a TβRI kinase inhibitor SB431542 (5 μM), a PI3K inhibitor LY294002 (10 μM), or 0.1% DMSO (vehicle-only control) for 1 h. Scale bars: 10 μm. Error bars represent SD (n = 5). *p* values were determined by Student's *t*-test. ∗*p* < 0.01. *D*, Rac1 activation assay using A549 cells pretreated with LY294002, SB431542, or 0.1% DMSO, followed by stimulation with 1 ng/ml TGF-β1 for 12 h. Cell lysates were subsequently prepared and subjected to a Rac1 activation assay as in (*A*). *E*, TGF-β-induced phosphorylation of Akt (S473) in A549-S3KO cells expressing Smad3(RA/KP). Cells were pretreated with either 10 μM LY294002 or 0.1% DMSO for 1 h and stimulated with 1 ng/ml TGF-β1 for either 1 h or 4 h. Cell lysates were analyzed by immunoblotting using phospho-Akt or phospho-Smad2 antibodies; α-tubulin was used as a loading control. *F*, effects of a ROCK inhibitor Y27632 on TGF-β-induced Rac1 activation in A549-S3KO expressing either Smad3 or Smad3(RA/KP). Cells were treated with TGF-β (1 ng/ml) and/or Y27632 (25 μM) for 12 h on collagen-coated plate. Cell lysates were subsequently prepared and subjected to a Rac1 activation assay as in (*A*). One representative result from two independent experiments is shown (*B–F*).

Because lamellipodia formation is dependent on the active form of Rac1, a small G protein of the Rho family (38, 39), we next examined a time course of Rac1 activation status after TGF-β stimulation in A549 cells. Rac1 was activated 8–12 h after stimulation (Fig. 6*A*). Then we examined the Rac1 activation in A549-S3KO cells, in which either wild-type Smad3 or Smad3(RA/KP) was expressed, after TGF-β stimulation for 12 h. Smad3(RA/KP) failed to rescue Rac1 activation (Fig. 6*B*).

Rac1 is activated by a variety of cytokines and growth factors. One of the pathways leading to Rac1 activation involves PI3K and its downstream Rac-GEFs including Vav1 and Vav2, which are derepressed by binding to phosphatidylinositol 3,4,5-tris phosphate (PIP_3_) *via* their pleckstrin homology domains (24). As previously reported for other cell lines (23, 40), a pharmaceutical PI3K inhibitor blunted TGF-β-induced cell motility and Rac1 activation in A549 cells (Fig. 6, *C* and *D*). One possible explanation is that Smad3(RA/KP) fails to rescue some process in the PI3K-Rac1 axis. TGF-β is known to activate PI3K *via* the TRAF6 pathway (9) in a manner dependent on the type I receptor kinase (41) but not on Smad3 in A549 cells, as assessed by PIP_3_-dependent phosphorylation of Akt at Ser-473 (Fig. S4). Consistently, TGF-β-induced Akt phosphorylation at Ser-473 was not attenuated in A549-S3KO cells expressing Smad3(RA/KP), suggesting successful formation of PIP_3_ that derepresses Rac-GEFs to activate Rac1 (Fig. 6*E*).

We further examined the effect of inhibition of Rac-GAPs on Rac1 activation in A549-S3KO cells expressing Smad3(RA/KP). Rho-associated kinase (ROCK) has been shown to activate Rac-GAPs (42, 43, 44). Therefore Y27632, a ROCK inhibitor, was used to downregulate active Rac-GAPs. In A549-S3KO cells expressing Smad3, Rac1 was activated by TGF-β to the equivalent level irrespective of the presence of Y27632. In A549-S3KO cells expressing Smad3(RA/KP), Rac1 was activated by TGF-β in the presence of Y27623, but not in the absence of Y27623 (Fig. 6*F*). We therefore hypothesized that Rac1 activation is not attenuated; instead, Rac1 inactivation is promoted in cells expressing Smad3(RA/KP).

### TGF-β downregulates ARHGAPs to sustain Rac1 activation

**Figure 7:**
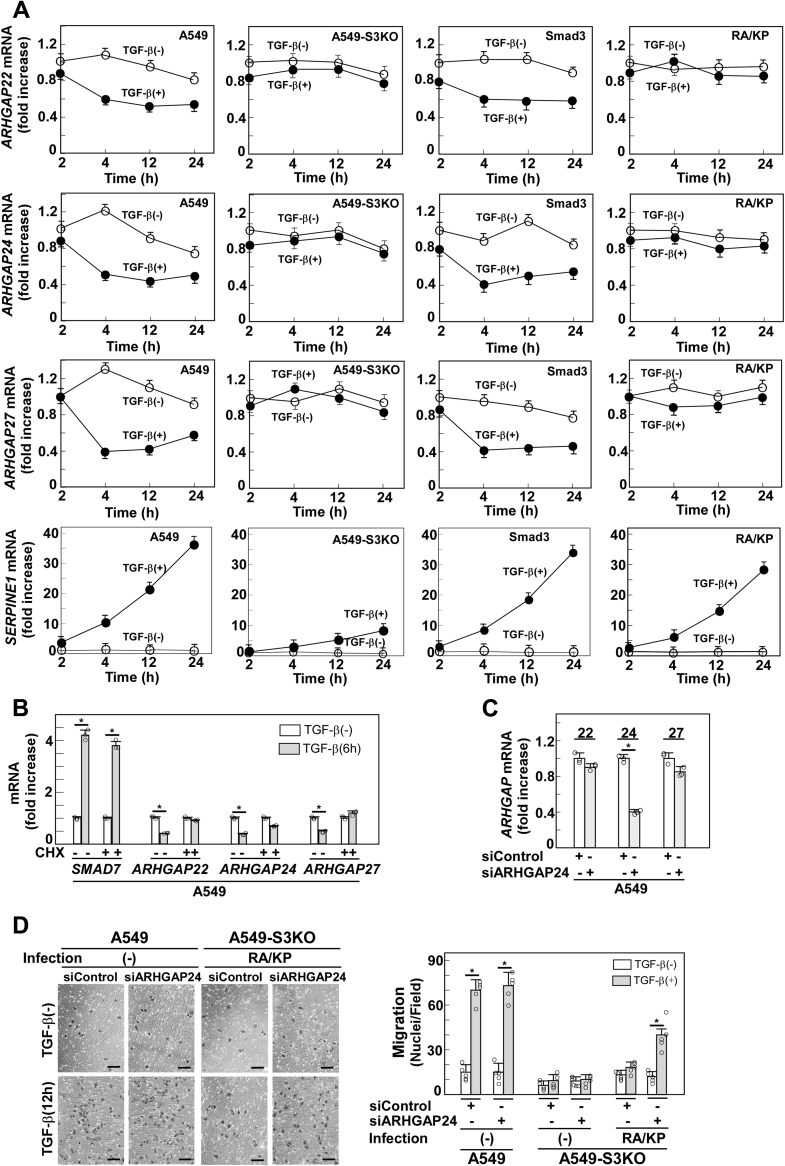
**Multiple *ARHGAP*s are downregulated during sustained Rac1 activation.***A*, TGF-β-induced downregulation of *ARHGAPs*. A549 cells, A549-S3KO cells, or A549-S3KO cells expressing either wild-type Smad3 or Smad3(RA/KP) were stimulated with 1 ng/ml TGF-β1 for 2–24 h. Total RNAs were extracted and analyzed by quantitative real-time PCR to determine endogenous *ARHGAP22*, *ARHGAP24*, *ARHGAP27*, and *SERPINE1* levels; *GAPDH* was used for normalization. *B*, effect of a protein synthesis inhibitor on downregulation of *ARHGAPs* by TGF-β. A549 cells pretreated with either 5 μM cycloheximide (CHX) or 0.1% DMSO for 1 h were stimulated with 1 ng/ml TGF-β1 for 6 h. *ARHGAP22*, *ARHGAP24*, *ARHGAP27*, or *SMAD7* mRNA expression was measured by quantitative real-time PCR. *SMAD7* is a direct target gene of TGF-β that is not affected by CHX. *C*, knockdown of *ARHGAP24* in A549 cells. Cells were treated with control siRNA (siControl) or *ARHGAP24* siRNA for 24 h. The expression level of mRNA was measured by quantitative real-time PCR. *D*, effect of *ARHGAP24* knockdown on TGF-β-induced cell motility in A549-S3KO cells expressing Smad3(RA/KP). Scale bars: 10 μm. Error bars represent SD (n = 3 for *A–C* or n = 5 for *D*). *p* values were determined by Student's *t*-test. ∗*p* < 0.01. One representative result from three (*A*) or two (*B–D*) independent experiments is shown.

To elucidate the mechanism underlying promoted Rac1 inactivation in cells expressing Smad3(RA/KP), we analyzed the transcriptome of A549-S3KO cells expressing either wild-type Smad3 or Smad3(RA/KP). Cells were stimulated with TGF-β for 8 h, a time point when Rac1 is sufficiently activated, before they were harvested for RNA-sequencing analysis. Genes regulated differently in the two cell types were clustered and subjected to pathway analysis. We noticed that genes associated with “GTPase activator activity” are significantly concentrated in a gene cluster that is downregulated in cells expressing wild-type Smad3 but not in cells expressing Smad3(RA/KP) (Fig. S5*A*). Among differently regulated genes in this cluster, we focused on *ARHGAP22*, *ARHGAP24*, and *ARHGAP27* (Fig. S5*B*). *ARHGAP* genes encode GTPase activating proteins (GAPs) that promote GTP hydrolysis to inactivate the Rho family of small G proteins (42). *ARHGAP22*, *ARHGAP24*, and *ARHGAP27* all use Rac1 as a substrate (42, 45, 46). Intriguingly, ARHGAP22 and 24 have been reported to be activated by ROCK1 (42, 43, 44). Time course measurement of *ARHGAP22*, *24*, and *27* expression indicates that they are downregulated from 4 h up to 24 h after TGF-β stimulation while not in A549-S3KO cells expressing Smad3(RA/KP) (Fig. 7*A*). The result is consistent with the time course of Rac1 activation (Fig. 6*A*). Furthermore, Smad3's β4 region-dependent downregulation of *ARHGAP24* and *ARHGAP27* was also observed in PANC-1 human pancreatic cancer cells (Fig. S6). Downregulation of *ARHGAP*s was inhibited by pretreatment with cycloheximide, indicating that they are indirect target genes of TGF-β, downregulation of which is dependent on protein synthesis (Fig. 7*B*). When we knocked down *ARHGAP2*4 in A549-S3KO cells expressing Smad3(RA/KP), TGF-β-enhanced cell motility was around 60% restored (Fig. 7, *C* and *D*). These results indicate that downregulation of *ARHGAP24* is one of the key events in enhancement of cell motility by TGF-β, but downregulation of other *ARHGAP*s could also contribute to the enhanced cell motility. Therefore, it appeared likely that TGF-β downregulates these *ARHGAP*s to prevent the inactivation of Rac1.

**Figure 8:**
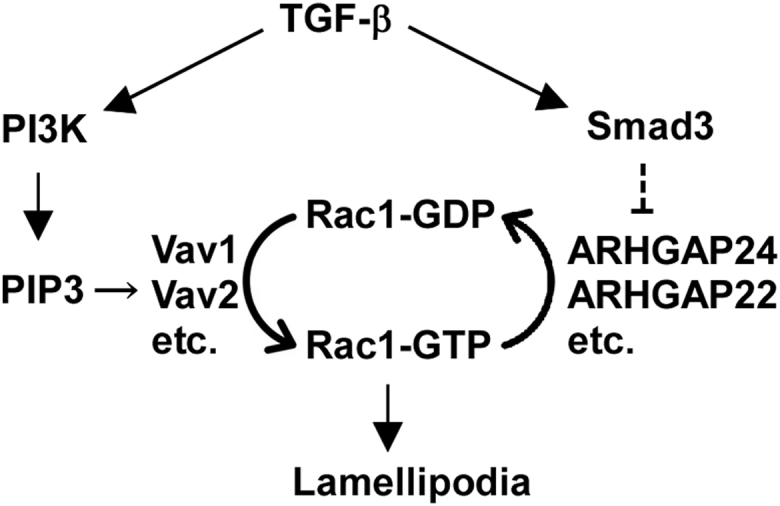
**Signaling pathway leading to Rac1 activation by TGF-β signaling**.

Thus, TGF-β induces sustained activation of Rac1 through PI3K-dependent Rac1 activation as well as through attenuation of Rac1 inactivation by downregulating multiple *ARHGAP*s (Fig. 8). The finding explains why TGF-β–induced cell motility requires a Smad-dependent transcriptional program in addition to PI3K activation.

## Discussion

The roles of TGF-β in cancer progression are well documented. Among the cellular responses induced by TGF-β in cancer cells, EMT is thought to promote cell invasion and metastasis (11). These cellular responses are primarily mediated by the coordinated actions of transcription factors associated with EMT (EMT-TFs) (12, 13). TGF-β activates multiple signaling pathways, including those that lead to induction of EMT-TFs and exert a series of EMT-associated cellular responses, some of which are modulated by pathways independent of EMT-TFs. Thus far, TGF-β-triggered signaling pathways that lead to induction of EMT-TFs have been characterized (15, 16, 17, 18, 19). However, the pathways that lead to individual cellular responses remain unclear. In this study, we identified the β4 region of Smad3's MH1 domain as a region indispensable for TGF-β-induced cell motility, but not epithelial marker downregulation, cell morphological changes, nor actin stress fiber formation.

A migrating cell exhibits high Rac1 activity at the leading edge, where Rac1 promotes the formation of membrane protrusions (47). We found that at least two independent signaling pathways are required to maintain Rac1 activation (Fig. 8): the PI3K-dependent pathway for Rac1 activation and another that is dependent on the β4 region of Smad3's MH1 domain, which attenuates Rac1 inactivation by repressing the expression of Rac1-targeting GAP proteins. This finding illustrates how the Smad3-dependent transcriptional program complements the well-known PI3K-dependent pathway in enhancing cell motility.

It remains unclear how the β4 region of Smad3 affects downregulation of a subset of target genes of TGF-β. As shown in Figure 7*B*, downregulation of *ARHGAP*s is dependent on *de novo* protein synthesis. Therefore, the involvement of miRNA processing by Smad3 may be excluded. We searched for Smad3-binding sites that are located near the *ARHGAP* genes in TGF-β-stimulated A549 cells using our previous ChIP-seq data GSM1246721 (48). We found no Smad3-binding sites within 100 kb upstream of the transcription start sites of *ARHGAP22* and *ARHGAP24*, nor 100 kb downstream of their transcription stop sites. There was, however, one strong Smad3-binding site 20 kb downstream of the *ARHGAP27* transcription stop site. This is consistent with our previous finding that Smad-binding sites are not often found within the regulatory regions of TGF-β–downregulated genes (49). In addition, the Smad3-binding region downstream of *ARHGAP27* failed to respond to TGF-β when introduced into a luciferase reporter vector pGL4-MLP (unpublished observation). Therefore, *ARHGAP*s are unlikely to be regulated by Smad3 binding to their regulatory regions. Although we observed the downregulation of *ARHGAP22*, *24*, and *27* in the present study, downregulation of other *ARHGAP*s that target Rac1 may also be involved in the process. However, they should be sensitive to a ROCK1 inhibitor because ROCK1 inhibitor was effective in restoring TGF-β-induced Rac1 activation in cells expressing Smad3(RA/KP).

Smad3 may interfere with the functions of other transcription factors by interacting physically with them *via* the β4 region to repress *ARHGAP*s. For example, Smad3 represses the AhR/Arnt transcription factor complex by competing with AhR and Arnt for their association (5). However, this mechanism is unlikely because Smad3(K36D/S37A), which cannot associate with the Smad-binding DNA element (30), fails to rescue cell motility (Fig. S7). Another possibility is that Smad3 interacts with some Smad cofactors through the β4 region and induces a protein or proteins to downregulate expression of *ARHGAP*s. In this case, proteins that downregulate *ARHGAP*s may include not only transcription repressors but also proteins that somehow inhibit the function of transcriptional activators for *ARHGAP*s. Smad cofactors that have been reported to be involved in TGF-β-induced cell motility or invasion include JunB in breast cancer cells (50), Olig1 in NMuMG cells (32), and Sox4 in nontransformed human mammary epithelial cells (51). Olig1 and Sox4 interact with Smad3 through the MH2 domain. JunB possibly interacts with Smad3 through its MH1 domain, like c-Jun (34). However, the c-Jun-binding region was mapped to the α2-helix region comprising Lys-40–Lys-44, which is distinct from the β4 region identified in this study. In addition, an effector protein downstream of JunB that facilitates cell motility was identified as Wnt7B, an external ligand (50). Therefore, these Smad cofactors are unlikely to be involved. Instead, some other unknown Smad cofactors may play a role in downregulating *ARHGAP*s. Alternatively, other mechanisms, including the destabilization of *ARHGAP* mRNAs, may be involved.

We previously reported that a basic helix-loop-helix transcription factor, Olig1, is a Smad cofactor that is involved in TGF-β–induced cell motility in NMuMG cells (32). Olig1 associates with Smad3 *via* the L3 loop in the Smad3 MH2 domain. However, we found that Olig1 is not expressed in A549 cells (unpublished observation). Thus, some other Smad cofactors(s) probably compensate for the role of Olig1 in A549 cells. Although we found that the β4 loop in the Smad3 MH1 domain signals for cell motility in A549 cells, the MH2 domain may also play a role in TGF-β–enhanced cell motility in A549 cells because peptide blockers derived from the MH2 domain inhibit TGF-β–induced cell motility (unpublished observation). Therefore, additional signaling pathways may operate during enhancement of cell motility. Supporting this notion, knockdown of *ARHGAP24* restored TGF-β–enhanced cell motility in A549-S3KO cells expressing Smad3(RA/KP), but not in A549-S3KO cells (Fig. 7*D*), indicating that Smad3 transmits some signal(s) in addition to that leading to downregulation of *ARHGAP*s .

TGF-β induces formation of actin stress fibers and changes in cell morphology beginning several hours after stimulation. We found that both of these changes are Smad3-dependent, but that the β4 region is not involved. Intriguingly, TGF-β–induced stress fiber formation requires PI3K signaling, cooperation between Smad signaling and the Rho family GTPases, Cdc42 and RhoA, as well as protein synthesis (20, 21, 23), while TGF-β–induced cell morphological changes are PI3K-independent (23). These findings indicate a bifurcation of signaling pathways leading to actin stress fiber formation and changes to cell morphology downstream of Smad3. Further investigation will uncover a complete picture of TGF-β signaling pathways that drive cell motility and other EMT-associated cellular responses.

## Experimental procedures

### Cell culture

A549 and PANC-1 cells were obtained from the American Type Culture Collection as previously described (15, 48) and authenticated by short tandem repeat analysis. Establishment of *SMAD3*-knockout A549 cells was described previously (5). PANC-1 cells were cloned and used as a parental cell line for *SMAD3* knockout using Double Nickase plasmid (catalog no. sc-4000069-NIC-2; Santa Cruz). Deletion/disruption of the target genes was confirmed by sequencing (Fig. S6A). All cells were maintained in Dulbecco's modified Eagle's medium (DMEM) containing 10% fetal bovine serum (FBS), 50 units/ml penicillin, and 50 μg/ml streptomycin.

### Reagents and antibodies

Human recombinant TGF-β1 was obtained from R&D Systems. Cycloheximide was purchased from Nacalai Tesque. SB431542 was obtained from Calbiochem. LY29400 was obtained from Sigma-Aldrich. Y27632 was purchased from FUJIFILM Wako Pure Chemical Co. Rhodamine-phalloidin was purchased from Cytoskeleton, Inc. The following antibodies were also used: anti-Smad2/3 (610843, BD Bioscience); anti-phospho-Smad1 (9511, Cell Signaling Technology); anti-phosho-Smad2 (138D4, Cell Signaling Technology); anti-phosho-Smad3 (9520, Cell Signaling Technology); anti-Smad4 (B-8, Santa Cruz Biotechnology); anti-E-cadherin (610182, BD Bioscience); anti-FLAG (M2; Sigma-Aldrich); anti-Rac1 (23A8; Millipore); anti-phospho-Akt (9271, Cell Signaling Technology); and anti-α-tubulin (DM1A) (T9026, Sigma-Aldrich). Horseradish peroxidase (HRP)-conjugated goat anti-mouse IgG (115-035-003, Jackson Immuno Research) and goat anti-rabbit IgG (111-035-003, Jackson Immuno Research) were used as secondary antibodies.

### Biochemical assays

Cell lysis and immunoblotting were performed as previously described (32). Luciferase assay and quantitative real-time PCR were performed in triplicates as previously described (52). Error bars represent SD. Primer sequences are shown in Table S2. DNA affinity precipitation assay using 3xCAGA probe was performed as described (52).

### Immunofluorescence and actin staining

Immunofluorescence labeling of E-cadherin was performed as previously described (53). Actin stress fiber formation was detected by staining with Rhodamine-conjugated phalloidin as previously described (54).

### Smad1/3 chimeras and Smad3 mutant proteins

Smad1/3 chimeras, 1-3-3 that contains the MH1 domain from Smad1 and the linker and MH2 domain from Smad3 (residues 1-144 of Smad3 was replaced by those of Smad1), and 3-3-1(HD/RT) that contains the MH1 domain and the linker from Smad3 and the MH2 domain from Smad1 (residues Glu-239 to Ser-425 of Smad3 were replaced by Glu-278 to Ser-465 of Smad1 with a double mutation His425Arg/Asp428Thr), were prepared using a PCR-based approach (30). For Smad3 MH1 mutants, the MH1 domains of Smad1 and Smad3 were divided into five regions (regions 1–5). Each of these regions was exchanged between the two proteins. Region 1, residues 1–44; Region 2, residues 45–65; Region 3, 98–103; Region 4, 104–108; Region 5, 109–144 in Smad1 and Smad3.

### Lentivirus production

Lentiviral vectors encoding either Smad3 or Smad3 mutants were generated by Gateway technology (Invitrogen). Lentivirus particles were produced as previously described (15). Cells infected with Smad3, 3-3-1(HD/RT), Smad3(R/K), or Smad3(RA/KP) were cloned by limiting dilution in 96-well plates and multiple clones were used for experiments. Cells infected with other constructs were used as cell pools. As for cells infected with Smad3, we had difficulty in obtaining clones with exogenous Smad3 expression equivalent to endogenous Smad3 expression in parental A549 cells. Therefore we selected a clone with the lowest expression of Smad3 among ten clones.

### Cell motility assay

Cell motility was measured and quantified by chamber migration assays using a Cell Culture Insert with polyethylene terephthalate filters (8 μm pore size, Falcon) in 24-well plates as previously described (22). The filters were coated with collagen (Cellmatrix Type 1-C, Nitta Gelatin). Briefly, cells were dissociated with trypsin and seeded in the upper chamber at 4 × 10^4^ cells/well density in either the presence or absence of 1 ng/ml TGF-β in both upper and lower chambers. The chambers were incubated at 37 °C under 5% CO_2_ for 12 h. Wound closure assay was performed as previously described (53).

### Cell proliferation assay

Cells were seeded at 1 × 10^5^ /well in six-well plates and stimulated with TGF-β1 (1 ng/ml) at day-1. Cells were trypsinized and harvested at the indicated time points, and counted using a hemocytometer.

### Rac1 activation assay

Rac1 activation assay was previously described (54). In brief, 2 × 10^5^ cells were seeded on collagen-coated six-well plates (Cellmatrix Type 1-C), cultured for the indicated time in either the presence or absence of 1 ng/ml TGF-β1, and harvested. Rac1 activation was determined by a GST pulldown assay using a Rac1/Cdc42 activation assay kit (Millipore 17-441).

### RNA-sequencing (RNA-Seq)

To mimic the situation in a chamber migration assay, A549-S3KO cells infected with lentivirus carrying cDNA encoding either Smad3 or Smad3 RA/KP were trypsinized, seeded on plates coated with collagen (Cellmatrix Type 1-C), and simultaneously stimulated with 1 ng/ml TGF-β for 8 h. Total RNA was purified using RNeasy Plus (Qiagen). RNA-Seq was performed as described previously (55). Reads were aligned against the human genome (GRCh38/hg38) using HISAT2 (http://daehwankimlab.github.io/hisat2/). Normalized gene expression data was generated using StringTie (http://ccb.jhu.edu/software/stringtie/index.shtml) without *de novo* transcript assembly. The raw sequence data are available from the GENE Expression Omnibus under accession number GSE152015. Gene Ontology analysis of the RNA-Seq data was performed using iDEP 0.91 web tool (http://bioinformatics.sdstate.edu/idep/). Log_2_ fold-change values of TGF-β (8 h)/TGF-β (-) in Smad3 wild-type or Smad3(RA/KP) were uploaded, then top-12000 most highly variable genes were divided into 4 clusters. The enrichment analysis based on Gene Ontology (GO term Biological process) was performed.

### RNA interference

Transfection of siRNA into cells (2 × 10^5^) was performed using Lipofectamine RNAiMAX transfection reagent (Invitrogen). The targeting sequence of Stealth RNAi siRNA was as follows; human *ARHGAP24*, 5′- AAGAUAGAGUAUGAGUCCAGGAUAA-3′. The final concentration of the siRNA used was 5 nM.

### Statistical analysis

A two-sided Student's *t*-test was used to determine the significance of differences among experimental groups.

## Data availability

RNA-seq data were deposited in the Gene Expression Omnibus under the accession number GSE152015.

## Supporting information

This article contains supporting information.

## Conflict of interest

The authors declare that they have no conflicts of interest with the contents of this article.

## References

[1] Shi Y., Massagué J. (2003). Mechanisms of TGF-β signaling from cell membrane to the nucleus. Cell.

[2] Ross S., Hill C.S. (2008). How the Smads regulate transcription. Int. J. Biochem. Cell Biol..

[3] Yoon J.H., Sudo K., Kuroda M., Kato M., Lee I.K., Han J.S., Nakae S., Imamura T., Kim J., Ju J.H., Kim D.K., Matsuzaki K., Weinstein M., Matsumoto I., Sumida T. (2015). Phosphorylation status determines the opposing functions of Smad2/Smad3 as STAT3 cofactors in TH17 differentiation. Nat. Commun..

[4] Saitoh M., Endo K., Minami M., Fukasawa A., Imamura T., Miyazawa K. (2016). STAT3 integrates cooperative Ras and TGF-β signals that induce Snail expression. Oncogene.

[5] Nakano N., Sakata N., Katsu Y., Nochise D., Sato E., Takahashi Y., Yamaguchi S., Haga Y., Ikeno S., Motizuki M., Sano K., Yamasaki K., Miyazawa K., Itoh S. (2020). Dissociation of the AhR-ARNT complex by TGF-β-Smad signaling represses *CYP1A1* gene expression and inhibits benze[a]pyrene-mediated cytotoxicity. J. Biol. Chem..

[6] Davis-Dusenbery B.N., Hata A. (2010). Mechanisms of control of microRNA biogenesis. J. Biochem..

[7] Lee M.K., Pardoux C., Hall M.C., Lee P.S., Warburton D., Qing J., Smith S.M., Derynck R. (2007). TGF-β activates Erk MAP kinase signalling through direct phosphorylation of ShcA. EMBO J..

[8] Sorrentino A., Thakur N., Grimsby S., Marcusson A., von Bulow V., Schuster N., Zhang S., Heldin C.-H., Landström M. (2008). The type I TGF-β receptor engages TRAF6 to activate TAK1 in a receptor kinase-independent manner. Nat. Cell Biol..

[9] Hamidi A., Song J., Thakur N., Itoh S., Marcusson A., Bergh A., Heldin C.-H., Landström M. (2017). TGF-β promotes PI3K-AKT signaling and prostate cancer cell migration through the TRAF6-mediated ubiquitylation of p85α. Sci. Signal..

[10] Bierie B., Moses H.L. (2006). Tumour microenvironment: TGFβ: The molecular Jekyll and Hyde of cancer. Nat. Rev. Cancer.

[11] Tsubakihara Y., Moustakas A. (2018). Epithelial-mesenchymal transition and metastasis under the control of transforming growth factor β. Int. J. Mol. Sci..

[12] Yang J., Antin P., Berx G., Blanpain C., Brabletz T., Bronner M., Campbell K., Cano A., Casanova J., Christofori G., Dedhar S., Derynck R., Ford H.L., Fuxe J., Garcia de Herreros A. (2020). EMT International Association (TEMTIA). Guidelines and definitions for research on epithelial-mesenchymal transition. Nat. Rev. Mol. Cell Biol..

[13] Nieto M.A., Huang R.Y., Jackson R.A., Thiery J.P. (2016). EMT:2016. Cell.

[14] Nieto M.A. (2017). Context-specific roles of EMT programmes in cancer cell dissemination. Nat. Cell Biol..

[15] Horiguchi K., Shirakihara T., Nakano A., Imamura T., Miyazono K., Saitoh M. (2009). Role of Ras signaling in the induction of snail by transforming growth factor-β. J. Biol. Chem..

[16] Shirakihara T., Saitoh M., Miyazono K. (2007). Differential regulation of epithelial and mesenchymal markers by δEF1 proteins in epithelial-mesenchymal transition induced TGF-β. Mol. Biol. Cell.

[17] Su J., Morgani S.M., David C.J., Wang Q., Er E.E., Huang Y.H., Basnet H., Zou Y., Shu W., Soni R.K., Hendrickson R.C., Hadjantonakis A.K., Massagué J. (2020). TGF-β orchestrates fibrogenic and developmental EMTs via the RAS effector RREB1. Nature.

[18] Thuault S., Valcourt U., Petersen M., Manfioletti G., Heldin C.-H., Moustakas A. (2006). Transforming growth factor-β employs HMGA2 to elicit epithelial-mesenchymal transition. J. Cell Biol..

[19] Vincent T., Neve E.P., Johnson J.R., Kukalev A., Rojo F., Albanell J., Pietras K., Virtanen I., Philipson L., Leopold P.L., Crystal R.G., de Herreros A.G., Moustakas A., Pettersson R.F., Fuxe J. (2009). A SNAIL1-SMAD3/4 transcriptional repressor complex promotes TGF-β mediated epithelial-mesenchymal transition. Nat. Cell Biol..

[20] Edlund S., Landstrom M., Heldin C.-H., Aspenstrom P. (2002). Transforming growth factor-β-induced mobilization of actin cytoskeleton requires signaling by small GTPases Cdc42 and RhoA. Mol. Biol. Cell.

[21] Vardouli L., Vasilaki E., Papadimitriou E., Kardassis D., Stournaras C. (2008). A novel mechanism of TGF-β-induced actin reorganization mediated by Smad and Rho GTPase. FEBS J..

[22] Ikushima H., Komuro A., Isogaya K., Shinozaki M., Hellman U., Miyazawa K., Miyazono K. (2008). An Id-like molecule, HHM, is a synexpression group-restricted regulator of TGF-β signaling. EMBO J..

[23] Bakin A.V., Tomlinson A.K., Bhowmick N.A., Moses H.L., Arteaga C.L. (2000). Phosphatidylinositol 3-kinase function is required for transforming growth factor β-mediated epithelial to mesenchymal transition and cell migration. J. Biol. Chem..

[24] Campa C.C., Ciraolo E., Ghigo A., Germena G., Hirsch E. (2015). Crossroads of PI3K and rac pathways. Small GTPases.

[25] BabuRajendran N., Palasingam P., Narasimhan K., Sun W., Prabhakar S., Jauch R., Kolatkar P.R. (2010). Structure of Smad1 MH1/DNA complex reveals distinctive rearrangements of BMP and TGF-β effectors. Nucleic Acids Res..

[26] Shi Y., Wang Y.F., Jayaraman L., Yang H., Massagué J., Pavietich N.P. (1998). Crystal structure of a Smad MH1 domain bound to DNA: Insights on DNA binding in TGF-β signaling. Cell.

[27] Chai J., Wu J.W., Yan N., Massagué J., Pavletich N.P., Shi Y. (2003). Features of a Smad3 MH1-DNA complex. Roles of water and zinc in DNA binding. J. Biol. Chem..

[28] Qin B.Y., Chacko B.M., Lam S.S., de Caestecker M.P., Correia J.J., Lin K. (2001). Structural basis of Smad1 activation by receptor kinase phosphorylation. Mol. Cell.

[29] Chacko B.M., Qin B.Y., Tiwari A., Shi G., Lam S., Hayward L.J., De Caestecker M., Lin K. (2004). Structural basis of heteromeric smad protein assembly in TGF-β signaling. Mol. Cell.

[30] Kusanagi K., Kawabata M., Mishima H.K., Miyazono K. (2001). α-Helix 2 in the amino-terminal Mad homology 1 domain is responsible for specific DNA binding of Smad3. J. Biol. Chem..

[31] Mizuide M., Hara T., Furuya T., Takeda M., Kusanagi K., Inada Y., Mori M., Imamura T., Miyazawa K., Miyazono K. (2003). Two short segments of Smad3 are important for specific interaction of Smad3 with c-Ski and SnoN. J. Biol. Chem..

[32] Motizuki M., Isogaya K., Miyake K., Ikushima H., Kubota T., Miyazono K., Saitoh M., Miyazawa K. (2013). Oligodedrocyte transcription factor 1 (Olig1) is a Smad cofactor involved in cell motility induced by transforming growth factor-β. J. Biol. Chem..

[33] Lo R.S., Chen Y.G., Shi Y., Pavletich N.P., Massagué J. (1998). The L3 loop: A structural motif determining specific interactions between SMAD proteins and TGF-β receptors. EMBO J..

[34] Qing J., Zhang Y., Derynck R. (2000). Structural and functional characterization of the transforming growth factor-β-induced Smad3/c-Jun transcriptional cooperativity. J. Biol. Chem..

[35] Liu T., Zhao M., Liu J., He Z., Zhang Y., You H., Huang J., Lin X., Feng X.H. (2017). Tumor suppressor bromodomain-containing protein 7 cooperates with Smads to promote transforming growth factor-β responses. Oncogene.

[36] Grocott T., Frost V., Maillard M., Johansen T., Wheeler G.N., Dawes L.J., Wormstone I.M., Chantry A. (2007). The MH1 domain of Smad3 interacts with Pax6 and represses autoregulation of the Pax6 P1 promoter. Nucleic Acids Res..

[37] Yamazaki D., Kurisu S., Takenawa T. (2005). Regulation of cancer cell motility through actin reorganization. Cancer Sci..

[38] Ridley A.J., Paterson H.F., Johnston C.L., Diekmann D., Hall A. (1992). The small GTP-binding protein rac regulates growth factor-induced membrane ruffling. Cell.

[39] Nobes C.D., Hall A. (1999). Rho GTPases control polarity, protrusion, and adhesion during cell movement. J. Cell Biol..

[40] Hubchak S.C., Sparks E.E., Hayashida T., Schnaper H.W. (2009). Rac1 promotes TGF-β-stimulated mesangial cell type I collagen expression through a PI3K/Akt-dependent mechanism. Am. J. Physiol. Ren. Physiol..

[41] Duan D., Derynck R. (2019). Transforming growth factor-β (TGF-β)–induced up-regulation of TGF-β receptors at the cell surface amplifies the TGF-β response. J. Biol. Chem..

[42] Sanz-Moreno V., Gadea G., Ahn J., Paterson H., Marra P., Pinner S., Sahai E., Marshall C.J. (2008). Rac activation and inactivation control plasticity of tumor cell movement. Cell.

[43] Ohta Y., Hartwig J.H., Stossel T.P. (2006). FilGAP, a Rho- and ROCK-regulated GAP for Rac binds filamin A to control actin remodelling. Nat. Cell Biol.

[44] Nguyen L.K., Kholodenko B.N., von Kriegsheim A. (2018). Rac1 and RhoA: Networks, loops and bistability. Small GTPases.

[45] Müller P.M., Rademacher J., Bagshaw R.D., Wortmann C., Barth C., van Unen J., Alp K.M., Giudice G., Eccles R.L., Heinrich L.E., Pascal-Vargas P., Sanchez-Castro M., Brandenburg L., Mbamal G., Tucholska M. (2020). Systems analysis of RhoGEF and RhoGAP regulatory proteins reveals spatially organized RAC1 signalling from integrin adhesions. Nat. Cell Biol..

[46] Saito K., Ozawa Y., Hibino K., Ohta Y. (2012). FilGAP, a Rho/Rho-associated protein kinase-regulated GTPase-activating protein for Rac, controls tumor cell migration. Mol. Biol. Cell.

[47] Narumiya S., Tanji M., Ishizaki T. (2009). Rho signaling, ROCK and mDia1, in transformation, metastasis and invasion. Cancer Metastasis Rev..

[48] Isogaya K., Koinuma D., Tsutsumi S., Saito R.A., Miyazawa K., Aburatani H., Miyazono K. (2014). A Smad3 and TTF-1/NKX2-1 complex regulates Smad4-independent gene expression. Cell Res.

[49] Koinuma D., Tsutsumi S., Kamimura N., Taniguchi H., Miyazawa K., Sunamura M., Imamura T., Miyazono K., Aburatani H. (2009). Chromatin immunoprecipitation on microarray analysis of Smad2/3 binding sites reveals roles of ETS1 and TFAP2A in transforming growth factor β signaling. Mol. Cell. Biol..

[50] Sundqvist A., Morikawa M., Ren J., Vasilaki E., Kawasaki N., Kobayashi M., Koinuma D., Aburatani H., Miyazono K., Heldin C.-H., van Dam H., ten Dijke P. (2018). JUNB governs a feed-forward network of TGFβ signaling that aggravates breast cancer invasion. Nucleic Acids Res..

[51] Vervoort S.J., Lourenço A.R., Tufegdzic Vidakovic A., Mocholi E., Sandoval J.L., Rueda O.M., Frederiks C., Pals C., Peeters J.G.C., Caldas C., Bruna A., Coffer P.J. (2018). SOX4 can redirect TGF-β-mediated SMAD3-transcriptional output in a context-dependent manner to promote tumorigenesis. Nucleic Acids Res..

[52] Itoh Y., Koinuma D., Omata C., Ogami T., Motizuki M., Yaguchi S., Itoh T., Miyake K., Tsutsumi S., Aburatani H., Saitoh M., Miyazono K., Miyazawa K. (2019). A comparative analysis of Smad-responsive motifs identifies multiple regulatory inputs for TGF-β transcriptional activation. J. Biol. Chem..

[53] Ishii H., Saitoh M., Sakamoto K., Kondo T., Katoh R., Tanaka S., Motizuki M., Masuyama K., Miyazawa K. (2014). Epithelial splicing regulatory protein 1 (ESRP1) and 2 (ESRP2) suppress cancer cell motility via different mechanisms. J. Biol. Chem..

[54] Motizuki M., Saitoh M., Miyazawa K. (2015). Maid is a negative regulator of transforming growth factor-β-induced cell migration. J. Biochem..

[55] Kawasaki N., Miwa T., Hokari S., Sakurai T., Ohmori K., Miyauchi K., Miyazono K., Koinuma D. (2018). Long noncoding RNA NORAD regulates transforming growth factor-β signaling and epithelial-to-mesenchymal transition-like phenotype. Cancer Sci..

