# Supplementary material for: TGF-β-induced cell motility requires downregulation of ARHGAPs to sustain Rac1 activity
Source: J Biol Chem. 2021 Mar 17;296:100545. doi: 10.1016/j.jbc.2021.100545 (PMC8079281; doi:10.1016/j.jbc.2021.100545)
Supplement: Supplementary file 1 — Figures S1 to S7 and Tables S1 & S2 [file mmc1.pdf]

Supporting Information for

TGF-β–induced cell motility requires down-regulation of *ARHGAP*s to sustain Rac1 activity

Mitsuyoshi Motizuki, Daizo Koinuma, Takashi Yokoyama, Yuka Itoh, Chiho Omata, Kohei Miyazono, Masao Saitoh, and Keiji Miyazawa

Supplementary Figures: Fig. S1 to S7
Supplementary Table: Table S1 and S2

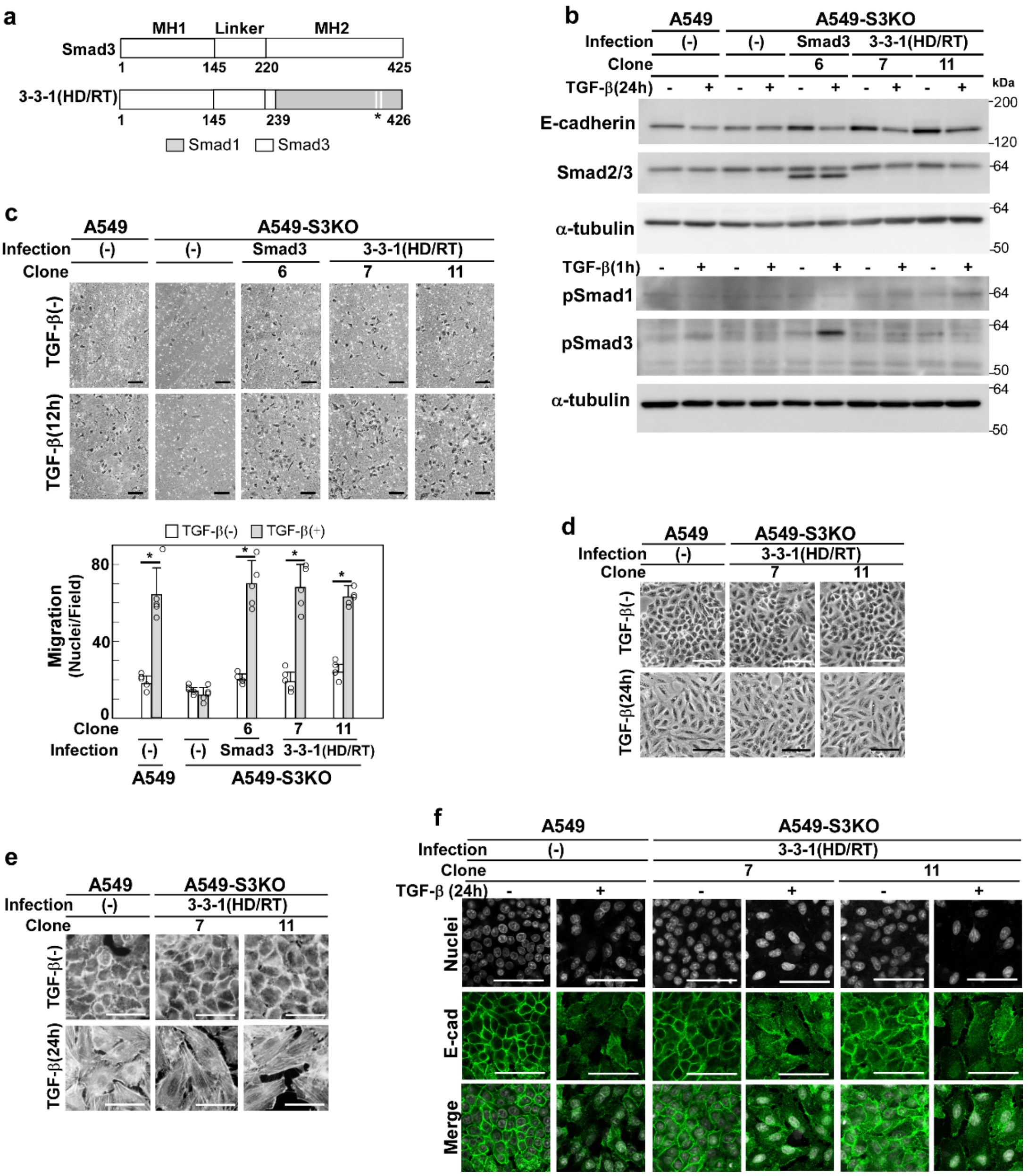

Fig. S1. Effects of Smad1/3 chimeric proteins in the MH2 domain on TGF-β-induced cell responses in A549 cells. **a** Schematic presentation of Smad3 and 3-3-1(HD/RT) chimeric protein (Smad3 with Glu-278 to Ser-465 from Smad1 with His425Arg/Asp428Thr substitution). The asterisk denotes the substituted site. The substitution allows C-terminal phosphorylation of the chimeric protein by TGF-β type I receptor. **b** A549-S3KO cells were infected with lentivirus carrying cDNA expressing 3-3-1(HD/RT) chimera and then subjected to cell cloning. Expression and TGF-β–induced phosphorylation as well as E-cadherin downregulation were verified by immunoblotting. **c** Chamber migration assay in A549-S3KO cells expressing 3-3-1(HD/RT) in either the presence or absence of 1 ng/ml TGF-β1. **d** Light microscopic images, **e** formation of actin stress fibers (Rhodamine-phalloidin staining), and **f** immunofluorescence detection of E-cadherin in A549-S3KO cells expressing 3-3-1(HD/RT) in either the presence or absence of 1 ng/ml TGF-β1. *Scale bars*: 10 µm. Error bars represent SD (n=5 for **c**). p values were determined by Student's t-test. *, $p < 0.01$. One representative result from two independent experiments is shown (**c**).

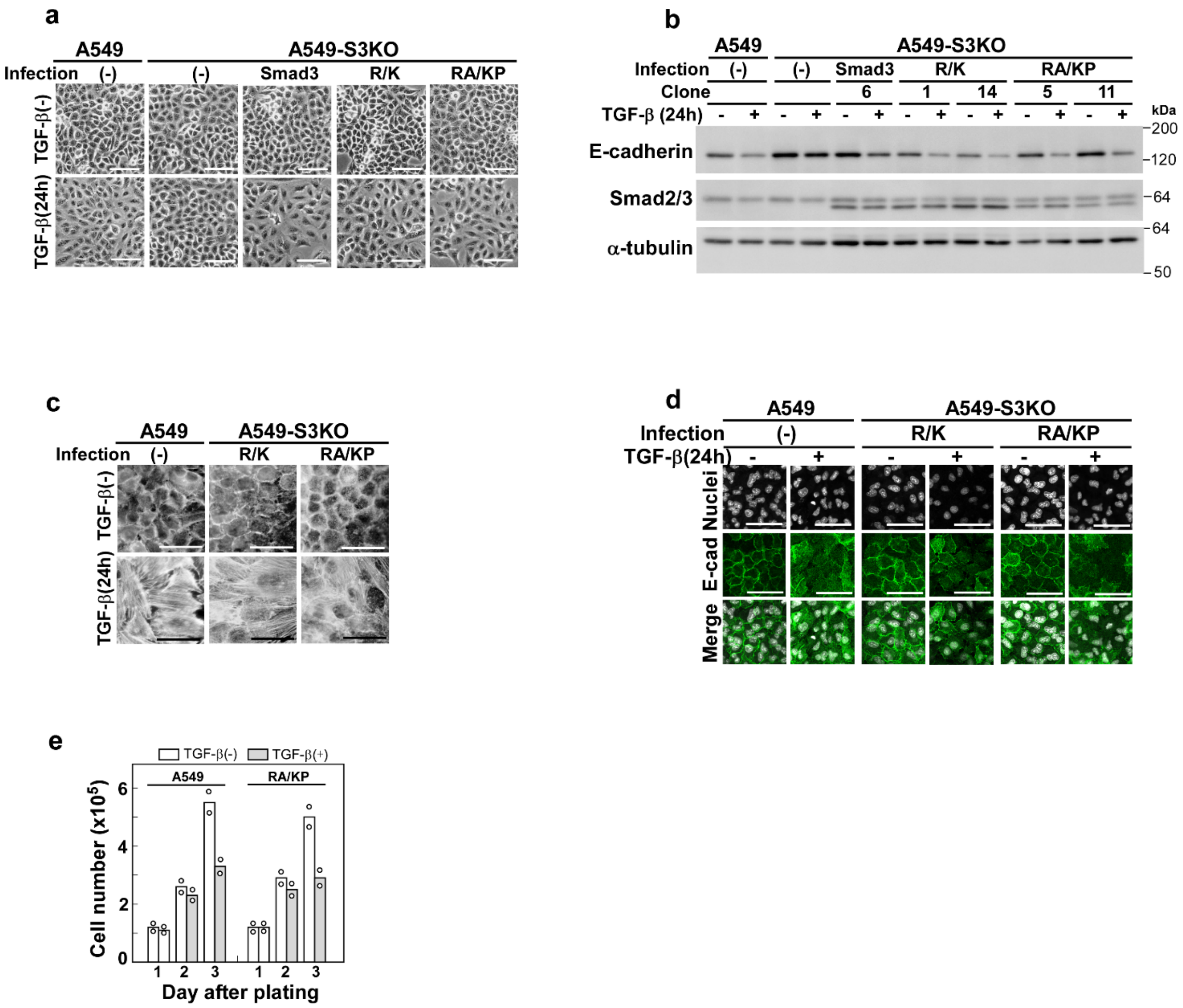

Fig. S2. Smad3(RA/KP) rescues EMT-associated responses other than cell motility. a Light microscopic images, **b** expression of E-cadherin (immunoblotting), **c** formation of actin stress fibers (Rhodamine-phalloidin staining), and **d** immunofluorescence detection of E-cadherin in A549-S3KO cells either expressing wild-type Smad3, Smad3(R/K), or Smad3(RA/KP) in either the presence or absence of 1 ng/ml TGF-β1. *Scale bars*: 10 μm. **e** Cell proliferation assay. One representative result from two independent experiments is shown (**e**).

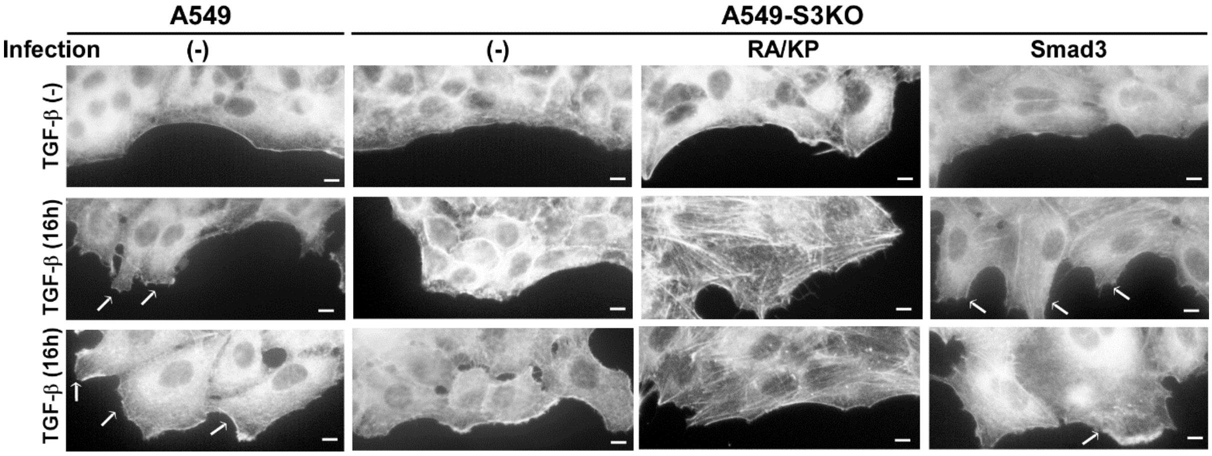

Fig. S3. Smad3(RA/KP) fails to rescue lamellipodia formation. Lamellipodia formation in TGF-β–stimulated cells. A549 cells were grown to confluence, scratched, and cultured for 16 h in either the presence or absence of 1 ng/ml TGF-β1. F-actin was stained using Rhodamine-phalloidin. Arrowheads indicate cells with lamellipodia. *Scale bars*: 1 μm.

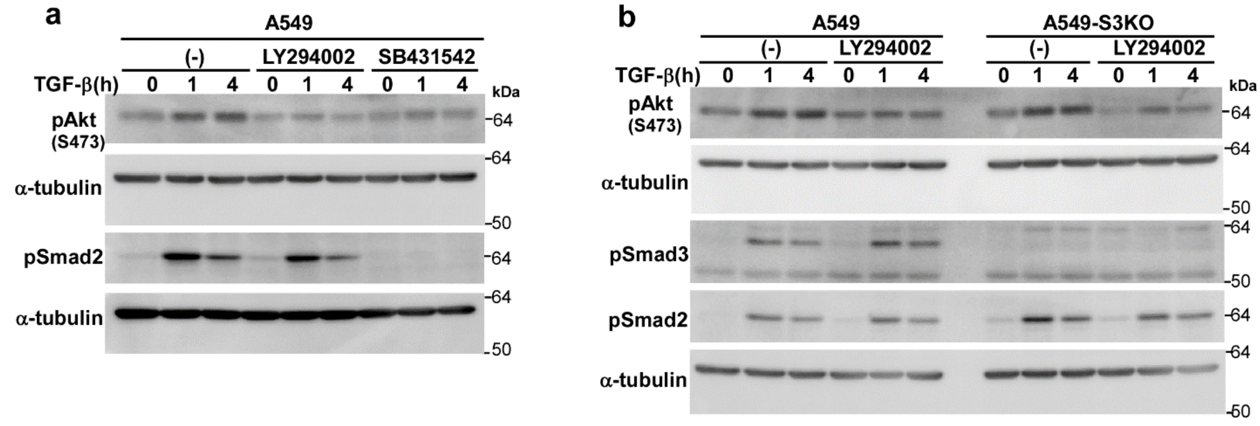

Fig. S4. PI3K activation by TGF-β is dependent on TβRI kinase activity but not on Smad3 in A549 cells. **a** Effect of a PI3K or TβRI kinase inhibitor on TGF-β–induced phosphorylation of Akt. A549 cells were treated with LY294002 (10 μM), SB431542 (5 μM), or 0.1% DMSO (vehicle) for 1 h and stimulated with 1 ng/ml TGF-β1 for 1 h or 4 h. Cell lysates were analyzed by immunoblotting using phospho-Akt or phospho-Smad2 antibodies. α-tubulin was used as a loading control. **b** TGF-β–induced phosphorylation of Akt in A549-S3 KO cells. The cells were treated with either LY294002 or 0.1% DMSO and TGF-β as in **a**. Cell lysates were analyzed by immunoblotting using indicated antibodies. α-tubulin was used as a loading control. One representative result from two independent experiments is shown (**a**, **b**).

a

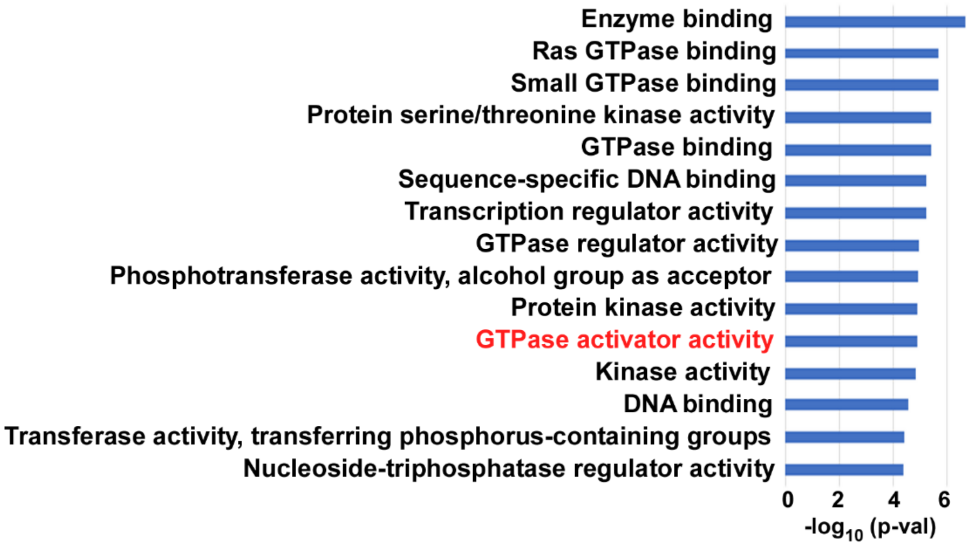

b

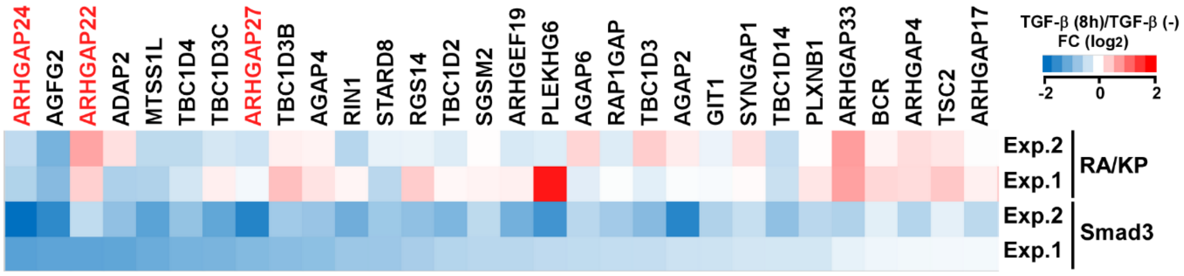

Fig. S5. Gene Ontology (GO) analysis of genes that are regulated differently by TGF-β in A549-S3KO cells expressing either wild-type Smad3 or Smad3(RA/KP). Cells were stimulated with TGF-β1 (1 ng/ml) for 8 h on collagen-coated plates, and harvested for RNA-sequencing. The relative expression ratio of TGF-β1 (8 h)/TGF-β1 (-) in cells expressing wild-type Smad3 was compared with that in cells expressing Smad3(RA/KP) in n = 2 independent experiments. **a** The most significant or enriched GO terms following K-means clustering in a gene cluster that is downregulated in cells expressing wild-type Smad3 but not in cells expressing Smad3(RA/KP). **b** Heat map showing genes associated with GTPase activator activity in TGF-β–stimulated A549-S3KO cells expressing Smad3 or Smad3(RA/KP).

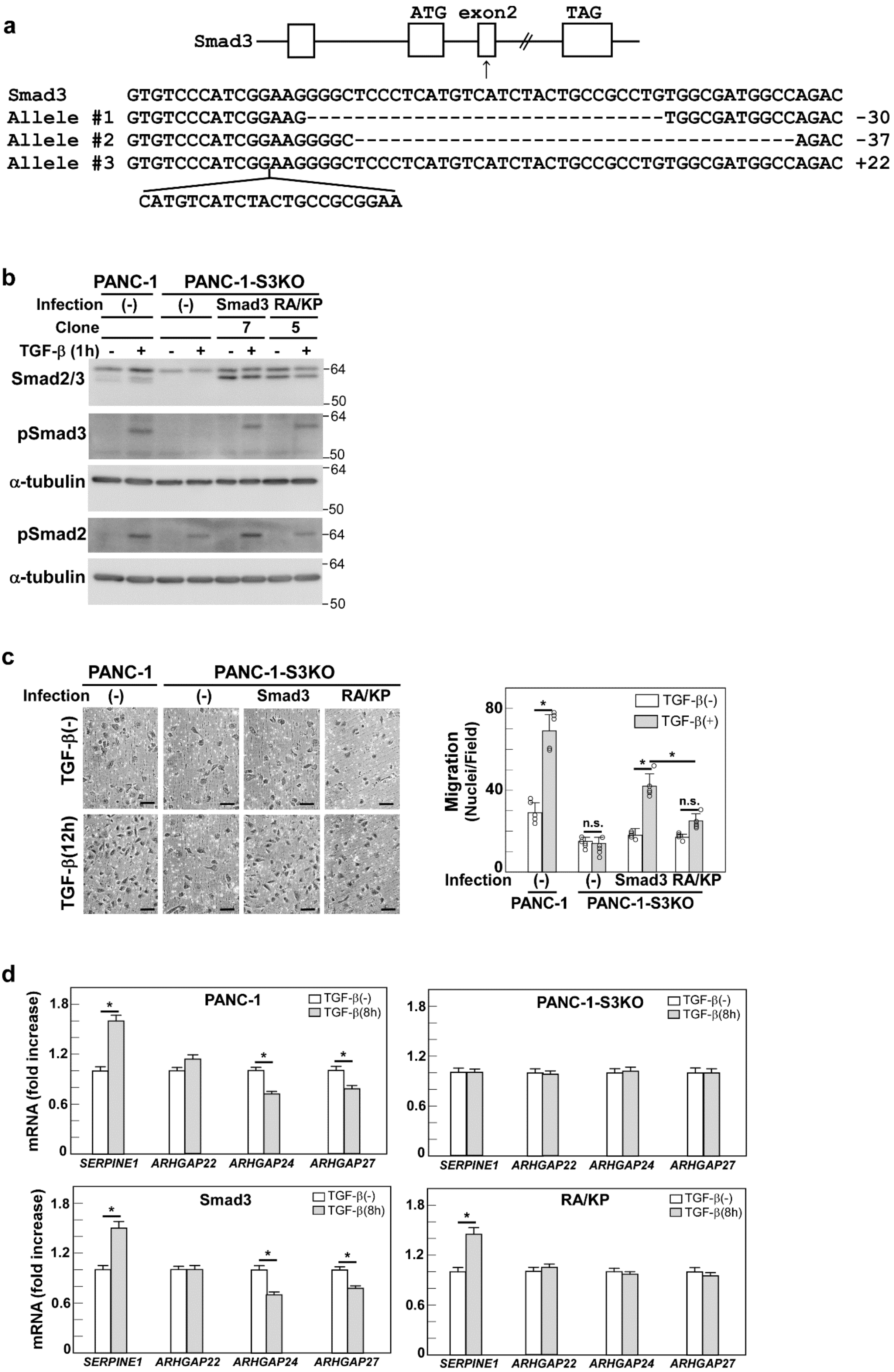
a
ATG
exon2
TAG
Smad3
Smad3 GTGTCCCATCGGAAGGGGCTCCCTCATGTCATCTACTGCCGCCTGTGGCGATGGCCAGAC
Allele #1 GTGTCCCATCGGAAG--------------------------------TGGCGATGGCCAGAC -30
Allele #2 GTGTCCCATCGGAAGGGGC-------------------------------------AGAC -37
Allele #3 GTGTCCCATCGGAAGGGGCTCCCTCATGTCATCTACTGCCGCCTGTGGCGATGGCCAGAC +22
CATGTCATCTACTGCCGCGGAA
b
PANC-1
PANC-1-S3KO
Infection (-) (-) Smad3 RA/KP
Clone 7 5
TGF-β (1h) - + - + - + - +
Smad2/3
pSmad3
α-tubulin
pSmad2
α-tubulin
64
50
c
PANC-1
PANC-1-S3KO
Infection (-) (-) Smad3 RA/KP
TGF-β(-)
TGF-β(12h)
TGF-β(-)
TGF-β(+)
Migration (Nuclei/Field)
80
40
0
n.s.
Infection (-) (-) Smad3 RA/KP
PANC-1 PANC-1-S3KO
d
PANC-1
PANC-1-S3KO
Smad3
RA/KP
TGF-β(-)
TGF-β(8h)
mRNA (fold increase)
1.8
1.2
0.6
0
SERPINE1
ARHGAP22
ARHGAP24
ARHGAP27

Fig. S6. Smad3-dependent downregulation of *ARHGAP*s in PANC-1 cells. a *SMAD3* knockout PANC-1 cells (PANC-1-S3KO) were prepared using CRISPR/Cas9-mediated genome editing. PANC-1 cells possess three alleles for *SMAD3* gene. All of Smad3 alleles in PANC-1-S3KO cells contain deletion or insertion in the exon2 of *SMAD3* gene as shown in allele #1 to #3. **b** PANC-1-S3KO cells were infected with lentivirus carrying cDNA encoding Smad3 or Smad3(RA/KP). Expression and TGF-β–induced phosphorylation of Smad3 were verified by immunoblotting using the indicated antibodies; α-tubulin was used as a loading control. **c** Chamber migration assay of PANC-1-S3KO cells expressing Smad3(RA/KP). Quantification is shown in the right. **d** TGF-β–induced downregulation of *ARHGAPs*. PANC-1 cells, PANC-1-S3KO cells, or PANC-1-S3KO cells expressing either wild-type Smad3 or Smad3(RA/KP) were stimulated with 1 ng/ml TGF-β1 for 8 h on collagen-coated plates and were subjected to quantitative real-time PCR. *Scale bars*: 10 μm. Error bars represent SD (n=5 for **c** and n = 3 for **d**). p values were determined by Student's t-test. *, $p < 0.01$. One representative result from two independent experiments is shown (**c**, **d**).

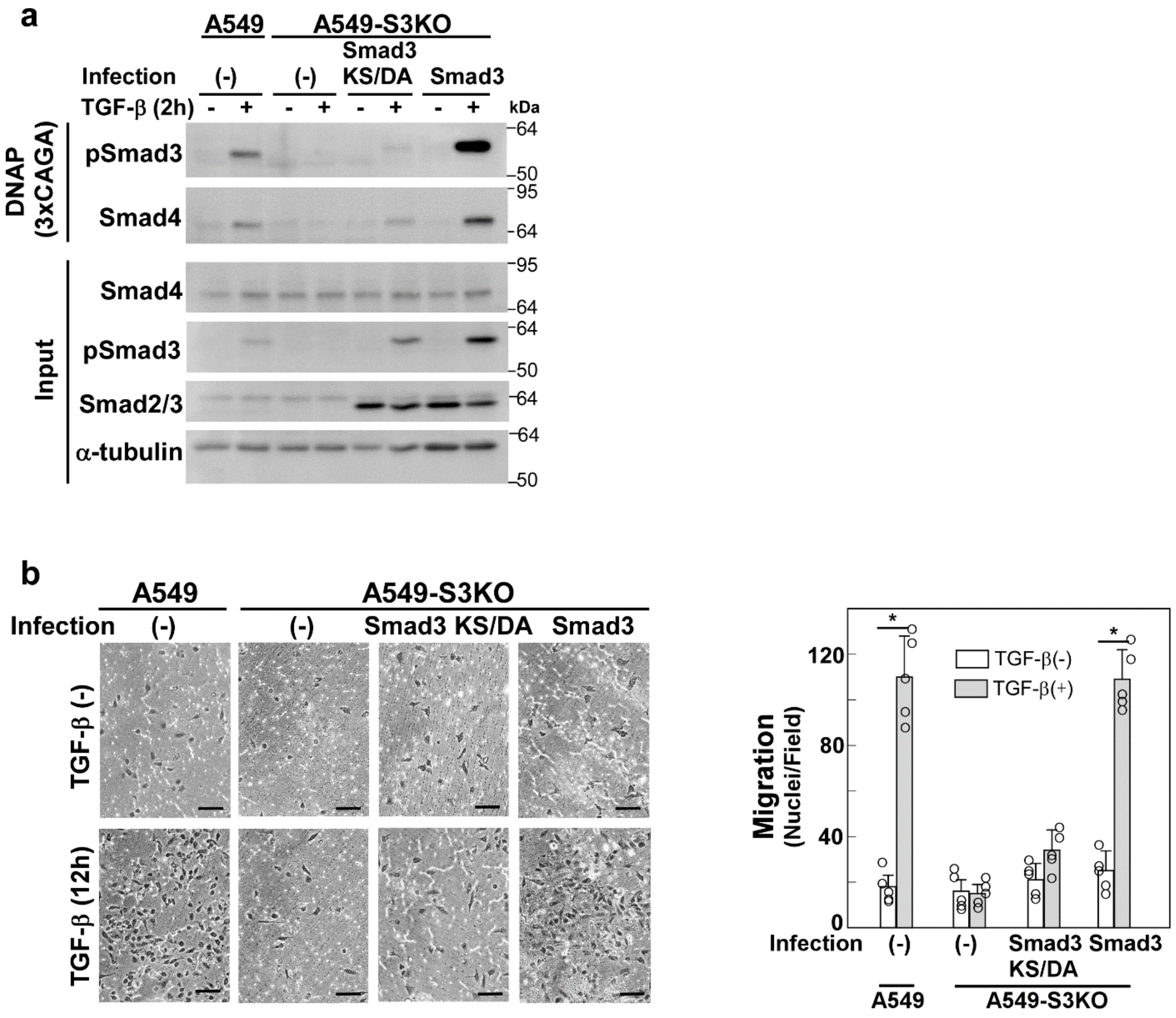

Fig. S7. Smad3 (KS/DA) fails to rescue TGF-β–induced cell motility. Smad3(KS/DA) is a mutant of Smad3 with both a Lys36Asp and Ser37Ala substitution; the two residues were replaced by the corresponding ones in Smad1. **a** DNAP assay using the 3xCAGA probe. **b** Chamber migration assay. *Scale bars*: 10 µm. Error bars represent SD (n = 5). *p* values were determined by Student's *t* test. *, $p < 0.01$. One representative result from two independent experiments is shown (**a**, **b**).

Table S1. Abilities of the Smad1/3 chimeras to support different cellular responses induced by TGF-β

	CAGA-Luc	DNAP	Cell motility	Morphological changes	Formation of stress fiber	Downregulation of E-cadherin
**N1133**	×	×	×	×	×	×
**N3133**	○		○	○	○	○
**N3311**	○	○	×	○	○	○
**N3311a**	○		×	○	○	○
**N3311b**	○		○	○	○	○
**RA/KP**	○		×	○	○	○

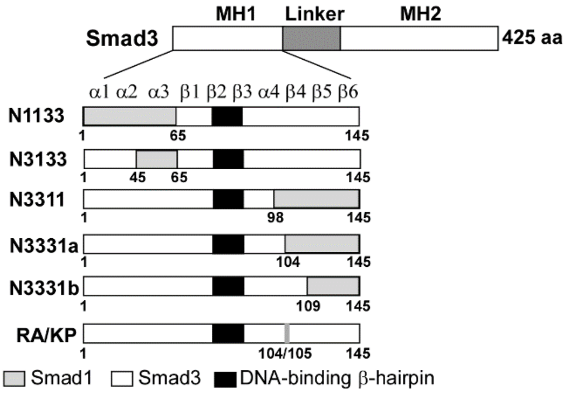

Table S2. Primers used for quantitative real-time PCR.

	Forward (5'→3')	Reverse (5'→3')
ARHGAP22	TCTGGCAACCGTTTTTGGAC	TGAAGAGCTGGCTGTGTTTG
ARHGAP24	TTGTGGCTGTGCTGTTTGTG	GCCTCGCAAAAGCAAAACTG
ARHGAP27	TTCACCAAGTGCACACCTTG	TTGAGCAAGTCACACTGCTG
GAPDH	GAAGGTGAAGGTCGGAGTC	GAAGATGGTGATGGGATTTC
SERPINE1	GGCTGACTTCACGAGTCTTTCA	ATGCGGGCTGAGACTATGACA
SMAD7	AAGCACCACCAAACACAGTG	ACAAAGAGCACGTTGTCTCC
